# Comparative Transcriptomics of Cold Growth and Adaptive Features of a Eury- and Steno-Psychrophile

**DOI:** 10.3389/fmicb.2018.01565

**Published:** 2018-07-31

**Authors:** Isabelle Raymond-Bouchard, Julien Tremblay, Ianina Altshuler, Charles W. Greer, Lyle G. Whyte

**Affiliations:** ^1^Department of Natural Resource Sciences, McGill University, Sainte-Anne-de-Bellevue, QC, Canada; ^2^Biotechnology Research Institute, National Research Council of Canada, Montreal, QC, Canada

**Keywords:** eurypsychrophile, stenopsychrophile, transcriptomics, cold growth, cold adaptation, permafrost, *Polaromonas*, *Rhodococcus*

## Abstract

Permafrost subzero environments harbor diverse, active communities of microorganisms. However, our understanding of the subzero growth, metabolisms, and adaptive properties of these microbes remains very limited. We performed transcriptomic analyses on two subzero-growing permafrost isolates with different growth profiles in order to characterize and compare their cold temperature growth and cold-adaptive strategies. The two organisms, *Rhodococcus* sp. JG3 (-5 to 30°C) and *Polaromonas* sp. Eur3 1.2.1 (-5 to 22°C), shared several common responses during low temperature growth, including induction of translation and ribosomal processes, upregulation of nutrient transport, increased oxidative and osmotic stress responses, and stimulation of polysaccharide capsule synthesis. Recombination appeared to be an important adaptive strategy for both isolates at low temperatures, likely as a mechanism to increase genetic diversity and the potential for survival in cold systems. While *Rhodococcus* sp. JG3 favored upregulating iron and amino acid transport, sustaining redox potential, and modulating fatty acid synthesis and composition during growth at -5°C compared to 25°C, *Polaromonas* sp. Eur3 1.2.1 increased the relative abundance of transcripts involved in primary energy metabolism and the electron transport chain, in addition to signal transduction and peptidoglycan synthesis at 0°C compared to 20°C. The increase in energy metabolism may explain why *Polaromonas* sp. Eur3 1.2.1 is able to sustain growth rates at 0°C comparable to those at higher temperatures. For *Rhodococcus* sp. JG3, flexibility in use of carbon sources, iron acquisition, control of membrane fatty acid composition, and modulating redox and co-factor potential may be ways in which this organism is able to sustain growth over a wider range of temperatures. Increasing our understanding of the microbes in these habitats helps us better understand active pathways and metabolisms in extreme environments. Identifying novel, thermolabile, and cold-active enzymes from studies such as this is also of great interest to the biotechnology and food industries.

## Introduction

The discovery of active, growing communities in polar habitats has given rise to much interest in studying the microbes that inhabit these regions and obtaining a greater understanding of the mechanisms and adaptive features that allow them to grow in cryoenvironments, generally defined as environments that exist continuously and predominantly at subzero temperatures. Microorganisms living in cryoenvironments must adapt to overcome numerous physiological and kinetic challenges, including decreased membrane fluidity, reduced diffusion and reaction rates, lower enzyme activity, stable secondary inhibitory DNA/RNA structures, and protein misfolding (Bakermans et al., 2012; De Maayer et al., 2014). Subzero environments, including permafrost and ice, are characterized by limited water availability, and liquid water in these habitats is believed to exist primarily in salty brine veins where increased solute and salt concentrations prevent freezing (Gilichinsky et al., 2005). As such, mechanisms must also exist to overcome osmotic stress and high salt concentrations.

Organisms isolated from these environments and capable of growth at low (≤0°C) temperatures have generally been classified as eurypsychrophiles (formerly psychrotrophs/psychrotolerant), as those organisms possess a broader growth range (Tmax > 20°C), and while capable of growth at very low temperatures (-15°C), they usually retain optimum growth rates at temperatures above 20°C (Feller and Gerday, 2003; Cavicchioli, 2006; Bakermans et al., 2012). In comparison, stenopsychrophiles (formerly “true psychrophiles”) exhibit narrower growth ranges (Tmax ∼20°C), and usually grow optimally at temperatures ranging between 5 and 15°C. We will use the term cold-adapted bacteria or psychrophile here to refer to both steno- and eury-psychrophiles. While it is true that eurypsychrophiles generally grow optimally at >20°C, they often exhibit growth over a wide range of temperatures and their low temperature minimum is often lower than stenopsychrophiles; indeed, almost all of the organisms reported to date capable of growth at -5°C and below are eurypsychrophiles (Bakermans et al., 2007; Rodrigues et al., 2008; Bergholz et al., 2009; Mykytczuk et al., 2013; Koh et al., 2017). This is an interesting observation and raises questions about the cold-adaptive properties and limitations of both types of cold-adapted bacteria.

The vast majority of studies into cold adaptation and growth, especially proteomes and transcriptomes, have focused heavily on eurypsychrophiles, and most of these, on above zero growth conditions (reviewed extensively in Kawamoto et al., 2017 and Raymond-Bouchard and Whyte, 2017). Comparatively little is known about stenopsychrophiles, especially in bacteria, whose members are more limited, and include *Colwellia psychrerythraea* and *Desulfotalea psychrophila* (Rabus et al., 2004; Methé et al., 2005). One characteristic of stenopsychrophiles that may allow for faster growth at lower temperatures is their ability to increase energy and carbon metabolism at these temperatures, when compared to higher suboptimal temperatures (Mock and Hoch, 2005; Mock et al., 2005; Hwang et al., 2008; Chong et al., 2011). These mechanisms are often downregulated in eurypsychrophiles at lower temperatures (Bergholz et al., 2009; Mykytczuk et al., 2013; Tribelli et al., 2015).

While both types of organisms have been studied for their cold-adaptive properties, almost no studies have directly compared the two types of psychrophiles (eury- and steno-psychrophiles) to elucidate their cold-adapted features and compare and contrast the different strategies that characterize their varied growth capabilities and optimums. We aim to increase our knowledge of cold and subzero growth in permafrost isolated eury- and steno-psychrophiles and gain a better understanding of their cold-adaptive strategies for growth at these temperatures. The eurypsychrophile *Rhodococcus* sp. JG3, isolated from permafrost (Goordial et al., 2016) in the McMurdo Dry Valley, Antarctica, is capable of growth from at least -5 to 30°C, and grows optimally at 20–25°C. *Polaromonas* sp. Eur3 1.2.1, isolated from permafrost in Eureka (Steven et al., 2008), Ellesmere Island, Nunavut, is a stenopsychrophile capable of growth from -5 up to 22°C and exhibits a faster generation time (g) at 10°C (3.1 days) than 22°C (3.4 days), and only slightly lower at 0°C (3.6 days) (Raymond-Bouchard et al., 2018). *Polaromonas* sp. Eur3 1.2.1 is a slow-growing, fastidious organism, and while growth down to -5°C has been detected on ½ R2A agar supplemented with 7% sucrose, so far no liquid media adequate for subzero growth has been found. In this study, our goal was to perform transcriptomic analyses of both organisms at their lower temperature limits of growth, 0 and -5°C, in addition to higher ≥20°C temperatures, in order to characterize and compare their low temperature growth and cold-adaptive strategies.

We chose to focus on potentially relevant transcriptomic changes in each organism during cold growth, as determined using RNA-seq, since our goal was to highlight cold-active pathways and genes of interest, which could be targets of future studies. We acknowledge that these organisms are evolutionarily distant. Our goal was to observe cold-adaptive changes and trends between evolutionarily different organisms. While very similar organisms (i.e., same genus) would allow us to narrow down very specific cold adaptations or changes in specific strains, our interest lies in beginning to elucidate adaptations on a larger scale, such as those that may be universal, compared to those that may be more specific to certain groups. Using evolutionarily different organisms allow us to investigate these potential differences by looking at each organism individually, in parallel, from a lower to higher temperatures and then looking for relative changes or trends between them.

## Materials and Methods

### Culturing and Growth Conditions

*Polaromonas* sp. Eur3 1.2.1 and *Rhodococcus* sp. JG3 were cultured on ½ R2A and R2A agar (BD Difco, Franklin Lakes, NJ, United States), respectively. For RNA extraction, liquid cultures of each strain were grown in biological triplicate to mid-late exponential phase on ½ R2A at 0 and 20°C (OD_600_ 0.165) for *Polaromonas* sp. Eur3 1.2.1, and in tryptic soy broth (TSB; BD Difco) supplemented with 5% salt and 5% sucrose at -5°C (OD_600_ 1.0) and 25°C (OD_600_ 2.0) for *Rhodococcus* sp. JG3. *Polaromonas* sp. Eur3 1.2.1 is a slow-growing organism with low cell densities, and as a result, several cultures had to be combined into one for each triplicate condition to compensate for the reduced amount of biomass obtained. It is challenging to identify adequate low temperature growth media for psychrophilic organisms. Ideally, we would have liked to grow both organisms on the same media. However, *Polaromonas* sp. Eur3 1.2.1 is a very fastidious organism and we have only been successful in growing the organism on ½ R2A. *Rhodococcus* sp. JG3, on the other hand grows much better on TSB at subzero temperatures and exhibits very slow growth rates on ½ R2A such that it was not feasible to perform this experiment on ½ R2A with *Rhodococcus* sp. JG3 at -5°C. We understand that some of the differences in transcriptional responses may be a result of differential growth media and have attempted to take this under consideration in our discussion. However, it is important to state that we are primarily looking at changes in one organism during growth at higher vs. lower temperatures. Subsequently, we make comparisons between the two organisms when general trends of interest can be seen.

### RNA Extraction and Library Preparation

RNA extraction was performed using the Direct-zol RNA MiniPrep Plus Kit (#R2070) from Zymo Research (Irvine, CA, United States) according to manufacturer’s instructions. RNA quantity and purity were checked using the Qubit 3.0 Fluorometer (Thermo Fisher Scientific, Waltham, MA, United States) and 2100 Bioanalyzer (Agilent Technologies, Santa Clara, CA, United States). Sequencing libraries were prepared using the Illumina (San Diego, CA, United States) TruSeq Stranded mRNA LT Sample Prep Kit as outlined in the manufacturer’s guide.

### Illumina Sequencing and Bioinformatics Analysis

Sequencing was performed by RNAseq on the Illumina MiSeq system using a Reagent Kit v3 150 cycles with a 2 × 75 bp paired-end configuration. Sequencing raw data (728 mega-bases) were processed through our metatranscriptomics bioinformatic pipelines. Read count summaries are provided for each sequencing library in **Supplementary Table S1**. Sequencing adapters were removed from each read and bases at the end of reads having a quality score less than 30 were cutoff (Trimmomatic v0.32) (Bolger et al., 2014) to generate quality controlled (QC) reads. QC-passed reads were mapped (BWA mem v0.7.10)^1^ (unpublished) against *Polaromonas* sp. Eur3 1.2.1 or *Rhodococcus* sp. JG3 genome references to obtain contig abundance profiles. *Polaromonas* sp. Eur3 1.2.1 and *Rhodococcus* sp. JG3 genomes were obtained from the Joint Genome Institute^2^, ID 2619618817 and 2529292502, respectively. Alignment files in bam format were sorted by read coordinates using samtools v1.1 (Li et al., 2009) and only properly aligned read pairs were kept for downstream steps. Each bam file (containing properly aligned paired-reads only) was analyzed for coverage of genes and contigs using bedtools (v2.17.0) (Quinlan and Hall, 2010) using corresponding gene models (gene coordinates on each contig for both genome references). Only paired-reads both overlapping their contigs or genes were considered for gene counts. Coverage profiles of each sample were merged to generate an abundance matrix (rows = contig, columns = samples) for which a corresponding counts per million abundance matrix (edgeR v3.10.2) (Robinson et al., 2009) was generated. According to our experimental design, differentially expressed genes were assessed with edgeR (v3.10.2) using its generalized linear model approach detailed by the authors (see section 3.2.3, page 29)^3^ with transcriptomics raw counts matrices as input. Genes having a log fold-change (logFC) ratio ≥| 1.5| and false discovery rate (FDR) <0.05 were considered as differentially expressed between the two temperatures in each organism. We did not directly compare *Rhodococcus* sp. JG3 and *Polaromonas* sp. Eur3 1.2.1 counts, rather we observed them individually in parallel and looked for relative differences at each organism’s reaction to cold-growth conditions. Metadata for all samples reported in this study are available in **Supplementary Table S1**. Raw sequence reads of the shotgun transcriptomics data were submitted to the Sequence Read Archive under BioProject ID PRJNA422596^4^.

### Data Analysis

Predicted COG and KEGG assignments for *Rhodococcus* sp. JG3 (ID 2529292502) and *Polaromonas* sp. Eur3 1.2.1 (ID 2619618817) were downloaded from the JGI IMG/ER website^5^, and used to obtain preliminary function and pathway assignments. These were then manually cross-referenced against available literature for the proteins discussed in the text to ensure function and pathway assignments are as accurate as possible. Focus was given to those genes with logFC ≥ 1.5 (FDR < 0.05) for which functional and pathway assignments could best be predicted based on homology assignments, conserved domains, and literature data. The volcano plot was created in R (v3.4.0) (R Core Team, 2017).

## Results And Discussion

**FIGURE 1:**
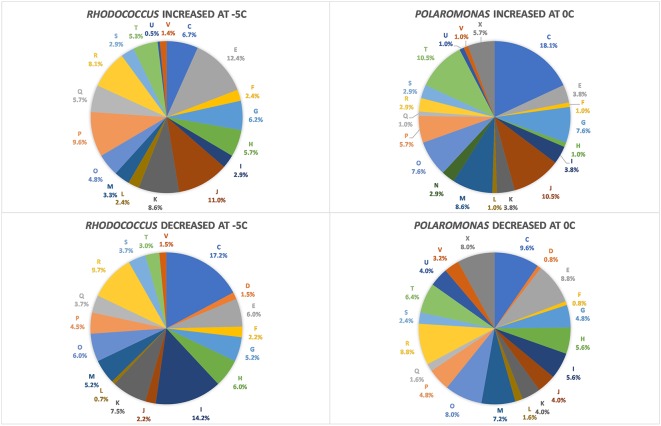
Diagram of COG categories with differentially expressed genes in *Polaromonas* sp. Eur3 1.2.1 at 0°C and *Rhodococcus* sp. JG3 at –5°C. The number of differentially expressed genes in each category is expressed as a percent of total. Letters correspond to COG categories: C, energy production and conversion; D, cell cycle control, cell division, and chromosome partitioning; E, amino acid metabolism and transport; F, nucleotide metabolism and transport; G, carbohydrate metabolism and transport; H, coenzyme metabolism and transport; I, lipid metabolism and transport; J, translation, ribosome structure, and biogenesis; K, transcription; L, replication, recombination, and repair; M, cell wall/membrane/envelope biogenesis; N, cell motility and secretion; O, post-translational modification, protein turnover, and chaperone functions; P, inorganic ion transport and metabolism; Q, secondary metabolites biosynthesis, transport, and catabolism; T, signal transduction; V, defense mechanisms; and R, general functional prediction only.

Using RNAseq we carried out transcriptomic analyses on (triplicate) cultures of *Rhodococcus* sp. JG3 grown at -5 and 25°C and *Polaromonas* sp. Eur3 1.2.1 grown at 0 and 20°C. Overall, 515 and 359 transcripts were found to be differentially expressed for *Rhodococcus* sp. JG3 and *Polaromonas* sp. Eur3 1.2.1, respectively, between their respective higher and lower temperatures (**Table 1** and **Supplementary Figure S1**). Of these about 66 and 45% could be assigned to COG and KEGG categories for both organisms. The largest shifts in abundance of transcripts observed for *Rhodococcus* sp. JG3 between -5 and 25°C, according to COG assignments, were in translation and ribosomal proteins, amino acid and inorganic ion transport and metabolism, lipid transport and metabolism, and energy production and conversion (**Figure 1**). Translation and amino acid and ion transport and metabolism processes saw significant upshifts in transcripts at -5°C, while transcripts involved in energy production and conversion, and lipid transport and metabolism were downregulated. Like *Rhodococcus* sp. JG3, *Polaromonas* sp. Eur3 1.2.1 also showed upregulation of transcripts involved in translation and ribosomal proteins at low temperature (0°C), compared to 20°C (**Figure 1**). However, unlike *Rhodococcus* sp. JG3, *Polaromonas* sp. Eur3 1.2.1 upregulated genes involved in energy production at low temperature and downregulated gene expression of amino acid transport and metabolism. In addition, noticeable increases in abundance of transcripts involved in signal transduction and carbohydrate transport and metabolism were also detected, along with a decrease in coenzyme transport and metabolism. Interestingly, at both 0 and 20°C *Polaromonas* sp. Eur3 1.2.1 upregulated a unique subset of transposon elements (discussed further below).

**Table 1:** Strain information and summary of transcriptomic results for *Rhodococcus* sp. JG3 and *Polaromonas* sp. Eur3 1.2.1.

	*Rhodococcus* sp. JG3	*Polaromonas* sp. Eur3 1.2.1
Phylum	Actinobacteria	Proteobacteria
Location isolated	Permafrost, University Valley, Antarctica	Permafrost, Eureka, Nunavut
Growth range	-5 to -30°C	-5^∗^ to -22°C
Salt (NaCl) tolerance	0–7%	0–3%
Size of genome (Mbp)	5.3	4.4
Total number of genes	5067	4303
Transcriptomic results		
Total differentially expressed genes (≥1.5 FC)	515	359
Increased at low temperature	313	177
Decreased at low temperature	202	182
With COG	343	236
% COG	67	66
With KEGG	234	161
% KEGG	45	45

^∗^-*5°C on agar only (Raymond-Bouchard et al., 2018).*

Overall, the response in *Polaromonas* sp. Eur3 1.2.1 was relatively more attenuated than in *Rhodococcus* sp. JG3. Fewer genes were found to be differentially regulated, with less than 40 genes upregulated >threefold change (logFC) at 0°C, and no transcripts with logFC > 5.8, as compared to *Rhodococcus* sp. JG3 where ∼150 genes were upregulated >threefold and up to 8.7 logFC (**Table 1** and **Supplementary Figure S1**). It is possible, given the reduced response and the fairly consistent generation times (3.6 days at 0°C, 3.1 at 10°C, and 3.4 days at 22°C) observed for *Polaromonas* sp. Eur3 1.2.1 over its temperature range that it remains somewhat constitutively adapted to the lower temperatures and does not need to shift processes to the extent that *Rhodococcus* sp. JG3 does at lower temperatures.

Regardless of the influence of different media used for the two organisms, differences between the cold and the optimal temperatures give us key insight into global transcriptional changes that can occur in an organism in response to cold. Our main aim was to compare the transcriptional differences in each organism from their higher to lower growth temperatures and to highlight potentially interesting changes that may be relevant to cold adaptation in each organism. Following this, in cases where differences could be observed between the organisms, we believed there was value in discussing these, especially since some of the differences observed are consistent with previous studies on steno- and eury-psychrophiles.

### Translation and Ribosomal Proteins

The importance of sustaining appropriate and necessary translation and ribosomal processes at low temperatures is apparent in the responses of both *Rhodococcus* sp. JG3 and *Polaromonas* sp. Eur3 1.2.1 at -5°C and 0°C, respectively. *Rhodococcus* sp. JG3 shows upregulation of at least 24 genes with logFC > 1.5 involved in translation and ribosomal processes, including numerous ribosomal proteins, a GTPase Era, the methylase RsmH, and the translation elongation factor EF-Ts (**Supplementary Table S2**) compared to the higher temperature 25°C. A queuine tRNA-ribosyltransferase and an *O*-acetyl-ADP-ribose deacetylase, which downregulates RNase III activity, as well as the ribosome binding factor A (*rbfA*) and a superfamily II DNA and RNA helicase (includes DEAD/DEAH-box family proteins) were upregulated >3 logFC. Helicases of this family are a common feature of cold growth in numerous cold-adapted bacteria, owing to their importance in stabilizing translation processes (Piette et al., 2011b). RbfA, involved in ribosome maturation and translation initiation, binds to the 30S subunit and is important for cold tolerance in *Escherichia coli*, where overexpression of this protein at cold temperatures (15°C) suppresses a cold-sensitive mutation and increases protein synthesis and growth (Dammel and Noller, 1995; Jones and Inouye, 1996). Upregulation of RbfA has also been reported for the psychrophiles *Exiguobacterium sibiricum* at -2.5°C (Rodrigues et al., 2008) and *Psychrobacter arcticus* 273-4 at -6°C (Bergholz et al., 2009). A fivefold upregulation of ADP-ribosylglycohydrolase, responsible for removal of ADP-ribose from ADP-ribosylated proteins was also observed in *Rhodococcus* sp. JG3 and may be important in the regulation of many cellular processes, including DNA/protein repair, DNA recombination, signal transduction, and gene transcription (Liu and Yu, 2015). Conversely, very few decreases were observed in this category (**Supplementary Tables S2**, **S4**) and included mostly translation inhibitors, such as the gene for an mRNA degradation ribonuclease and the ribosome-associated translation inhibitor RaiA, indicating the importance of keeping translation processes active at low temperatures.

Similarly, *Polaromonas* sp. Eur3 1.2.1 increased expression of transcripts for a number of ribosomal proteins, a superfamily II helicase, and the translation initiation factor IF-1, with concurrently small fold decreases in transcripts in this category (**Supplementary Tables S3, S5**) at 0°C compared to 20°C. Two of the most upregulated genes in *Polaromonas* sp. Eur3 1.2.1 were molecular chaperones, a disulfide isomerase subunit, *dsbG*, of the thiol:disulfide interchange protein, and a second thiol:disulfide isomerase. Another thiol:disulfide interchange subunit, *dsbD*, was also increased. The thiol:disulfide isomerases are required for proper folding of proteins through isomerization of incorrect disulfide bonds to the correct pattern (Shao et al., 2000; Gleiter and Bardwell, 2008). They prevent misfolding and aggregation of proteins, which is believed to be more prevalent at lower temperatures (Doyle et al., 2012). *Polaromonas* sp. Eur3 1.2.1 also downshifts expression of the Ribonuclease E in favor of its homolog Ribonuclease G, an enzyme with more limited enzymatic capabilities than RNase E (Deana and Belasco, 2004). The importance of sustaining translation at low temperatures has been highlighted in studies of both eury- and steno-psychrophiles (Goodchild et al., 2004; Mock et al., 2005; Nunn et al., 2015; Koh et al., 2017) and has been postulated to be the rate-limiting step for protein synthesis in certain cold communities (Toseland et al., 2013). Shifts in ribosomal and translation proteins ultimately allow the ribosome to become “cold-adapted” and preserve translation at cold temperatures, thus maintaining growth and metabolic activity.

### Transposons, DNA Recombination, and Genomic Redundancy

*Polaromonas* sp. Eur3 1.2.1 appears to express transcripts for a number of transposase elements, and at 0°C, shifts expression in favor of IS5 family transposases (**Supplementary Table S3**) compared to 20°C. IS5 can mediate directed mutations and causes gene activation or inactivation through insertion upstream of targeted genes near the promoter (Zhang and Saier, 2012). Transcripts for the competent protein, ComEA, important for free DNA uptake and horizontal gene transfer, was also increased slightly. While *Rhodococcus* sp. JG3 does not appear to differentially express transposon elements from -5 to 25°C, several genes with roles in DNA recombination, including a single strand DNA binding protein, the Holliday junction helicase (RuvA), and the alpha subunit (RecD) of exonuclease V were all increased ∼twofold at -5°C relative to 25°C (**Supplementary Table S2**). Two transposons and several recombination proteins were expressed in the psychrophilic archaeon *Methanococcoides burtonii* at 4°C (Goodchild et al., 2004) and several psychrophilic genomes, including the genome of subzero-growing *P. arcticus* 273-4, have been shown to possess large numbers of elements that contribute to genome plasticity, such as plasmids, transposons, and other mobile elements (Allen et al., 2009; Ayala-del-Río et al., 2010; Math et al., 2012). Recombination, both from transposition and site-specific recombination, may serve as a general adaptive strategy to increase genetic diversity and the potential for survival and growth in cold-temperature systems.

Indeed, genomic redundancy and isozyme exchange has been shown to be a cold-adaptive strategy in certain cold-adapted bacteria, including *E. sibiricum*, *P. arcticus*, *Planococcus halocryophilus*, and *Psychrobacter* sp. PAMC 21119, with different copies of genes being expressed at different temperatures (Rodrigues et al., 2008; Bergholz et al., 2009; Mykytczuk et al., 2013; Koh et al., 2017). Both *Rhodococcus* sp. JG3 and *Polaromonas* sp. Eur3 1.2.1 show evidence of genomic redundancy with high copy numbers of certain genes known to have roles during cold-temperature growth (Goordial et al., 2016; Raymond-Bouchard et al., 2018). In this study, differential expression was observed for several gene copies including catalase, the iron uptake regulator fur, sigma-70 polymerase, universal stress protein (uspA), TRAP dicarboxylate permease, and ABC-type polar amino acid transporter (**Table 2**) when comparing the higher and lower growth temperatures. This may confer an advantage during cold growth whereby cold-adapted versions of a given gene product would be expressed and likely function better under that condition.

**Table 2:** Genomically redundant differentially expressed genes in *Rhodococcus* sp. JG-3 and *Polaromonas* sp. Eur3 1.2.1.

*Rhodococcus* sp. JG-3	
Description, COG/KO, and Gene ID	logFC
*Acyl dehydratase*, *COG2030*	
2529298888/2529302388	-2.39/2.30
*Catalase-peroxidase katG*, *COG0376/K03782*	
2529300023/2529300594	-5.95/3.17
*ABC di-*, *oligo-peptide/nickel transport*, *permease*, *COG0601/K02033*	
2529299546/2529302471	-4.57/5.16
*Fe2+ or Zn2+ uptake regulation protein*, *fur*, *COG0735/K03711*	
2529300022/2529300593	-4.40/4.49
*DNA-binding transcriptional regulator*, *AcrR family*, *COG1309*	
2529299557/2529298884/2529299633	-5.36/-2.75/2.01
2529302950/2529299074/2529299715/2529301247	2.27/2.51/3.24/5.21
*RNA polymerase sigma-70 factor*, *ECF subfamily*, *COG1595/K03088*	
2529302703/2529303412	-1.91/5.51
*DNA-binding transcriptional regulator*, *MarR family*, *COG1846*	
2529302179/2529298833/2529299065	-2.27/2.96/5.73
*ADP-ribosylglycohydrolase*, *COG1397*	
2529302519/2529302299	-5.19/5.16
*Flavin-dependent oxidoreductase*, *luciferase family*, *COG2141*	
2529299256/2529298656	-4.56/-3.05
2529299395/2529300234/2529302665	2.55/2.76/4.73

***Polaromonas* sp. Eur3 1.2.1**	

**Description, COG/KO**	**logFC**

*2-keto-4-pentenoate hydratase/2-oxohepta-3-ene-1*,*7-dioic acid hydratase (catechol pathway)*, *COG0179*	
2619646842/2619645902/2619646695	1.81/-2.42/-3.12
*Tripartite-type tricarboxylate transporter*, *receptor*, *TctC*, *COG3181*	
2619647128/2619645167	5.16/2.40
2619646693/2619647684	-2.30/-4.11
*Thiol-disulfide isomerase or thioredoxin*, *COG0526*	
2619646415/2619646606	4.07/-2.54
*Nucleotide-binding universal stress protein*, *UspA family*, *COG0589*	
2619647233/2619644545/2619647313	3.42/-2.38/-5.54
*CRP cAMP-binding domain or regulatory unit cAMP kinases*, *COG0664*	
2619646293/2619647120/2619644878	2.95/2.23/-1.81
*ABC-type polar A.A. transport*, *periplasmic component*, *COG0834*	
2619645443/2619646238	2.10/-3.76
*TRAP-type C4-dicarboxylate transport*, *small permease*, *COG3090*	
2619646850/2619647206	2.32/-2.08

*logFC = log fold change as compared to the higher temperature culture. Fold changes (right column; logFC) for differentially expressed genes under each function description are separated by a forward slash and correspond to the given gene ID in the same order (left column).*

### Iron Acquisition

Fourteen genes with roles in iron acquisition and transport were found to be upregulated greater than 1.5-fold at -5°C in *Rhodococcus* sp. JG3 compared to 25°C, with 1/2 of these increased >5-fold (**Supplementary Table S2**). This response may be one way to counteract the reduced solubility of iron at cold temperatures (Lide, 2005). The most highly upregulated were those involved in the biosynthesis and transport of siderophores, including mycobactin (*mtbA*, *mtbB*, and *mtbI*) and hydroxamate siderophores (*pvdA*), as well as a ferric siderophore reductase likely involved in reduction-mediated release of the iron from the siderophore. Siderophores are low molecular weight molecules with very high affinity for ferric iron (Fe^3+^). The gene for the transcriptional repressor Fur, which binds ferrous iron (Fe^2+^) and negatively regulates iron uptake genes, was downregulated over fourfold (**Supplementary Table S2**). Fe^3+^ is a crucial co-factor in numerous cellular processes and the concurrently large increases in transcripts for genes containing or synthesizing porphyrin and Fe–S cluster containing molecules, such as protoporphyrinogen IX oxidase, catalases, selenocysteine dehydrogenase, and flavoproteins, explains the greater need for iron in *Rhodococcus* sp. JG3. Genes for iron uptake were not differentially expressed in *Polaromonas* sp. Eur3 1.2.1 at 0°C relative to 20°C, though a few genes for Fe–S cluster formation were slightly increased (1.5–2 logFC) (**Supplementary Table S3**). As with *Rhodococcus* sp. JG3, Fe–S clusters are important co-factors for a number of processes that are increased at cold temperature in *Polaromonas* sp. Eur3 1.2.1 including tetrathionate reductase and cytochromes important for energy generation.

Large increases in iron acquisition mechanisms are not usually observed in cold-adapted bacteria during cold-temperature growth, and there is evidence that iron uptake genes and iron-associated proteins are suppressed in cold-adapted bacteria (Piette et al., 2011a; Ronholm et al., 2015; Tribelli et al., 2015). This suppression is theorized to perhaps contribute to alleviating oxidative stress by iron (Fenton reaction). However, as with *Polaromonas* sp. Eur3 1.2.1, increases in Fe–S cluster generating proteins is one exception that has been reported (Goodchild et al., 2005; Bergholz et al., 2009). While this strategy of reliance on additional iron during cold growth in *Rhodococcus* sp. JG3 can be partly explained within the context of its natural environment, the McMurdo Dry Valleys, which have relatively high soluble (bioavailable) iron content and noticeable ferric oxides (Tamppari et al., 2012; Bhattachan et al., 2015), it is intriguing and warrants further investigation.

### Cell Envelope and Extracellular Polysaccharides

Some psychrophiles, such as *P. halocryophilus* and *Colwellia psychrerythraea*, create unique extracellular cell envelope structures at low temperatures, believed to offer some level of cryoprotection and antifreeze properties (Mykytczuk et al., 2013, 2015; De Maayer et al., 2014; Carillo et al., 2015). In *C. psychrerythraea*, this structure is a polysaccharide capsule with similarities to antifreeze proteins and glycoproteins. Both *Polaromonas* sp. Eur3 1.2.1 and *Rhodococcus* sp. JG3 increase the abundance of transcripts predicted to be in involved in exopolysaccharide (EPS) and capsular polysaccharide biosynthesis at their respective low temperatures compared to their respective higher temperatures. These included colanic acid biosynthesis, UDP-glucose lipid carrier transferase, cellulose synthase, EPS biosynthesis proteins, mannose-6-phosphate isomerase, and heparin binding hemagglutinin (**Supplementary Tables S2, S3**). The EPS biosynthesis protein is part of the EPS system in *Bacillus subtilis* (Roux et al., 2015), and heparin binding hemagglutinin induces aggregation of cells in mycobacteria (Menozzi et al., 1996). Mutational analyses to study impaired cold-temperature growth in the deep-sea bacterium *Photobacterium profundum*, found the largest fraction of genes associated with temperature sensitivity to be involved in the cell envelope formation, specifically extracellular polysaccharide biosynthesis (Lauro et al., 2008), implying that some level of EPS synthesis is especially relevant at cold temperatures. In addition, the capsule from *C. psychrerythraea* has been theorized to function as a protective mechanism at subzero temperatures (Carillo et al., 2015).

### Cell Wall/Membrane

A common feature of many cold-adapted microorganisms is the presence of a system to increase or modulate membrane flexibility at lower temperatures (De Maayer et al., 2014). There is evidence for membrane modifications in *Rhodococcus* sp. JG3 at -5°C compared to 25°C with significant upregulation of an acyl-CoA thioesterase, an important regulator of lipid metabolism involved in both synthesis and degradation of fatty acids (Hunt and Alexson, 2002), as well as an acyl dehydratase and a putative flippase (**Supplementary Table S2**). Flippases are important for maintaining asymmetric distributions of phospholipids, while acyl dehydratases can create double bonds in fatty acids. Increasing content of unsaturated fatty acids in the membrane is a common mechanism to increase flexibility in cold-adapted organisms (De Maayer et al., 2014). Similar increases were not observed in *Polaromonas* sp. Eur3 1.2.1 However, emphasis on increased peptidoglycan synthesis and remodeling at 0°C compared to 20°C was apparent in *Polaromonas* sp. Eur3 1.2.1 with a ∼twofold upregulation of transcripts for three enzymes, undecaprenyl diphosphate synthase and UDP-*N*-acetylmuramyl tripeptide synthase (*murE*), vital for peptidoglycan synthesis, and membrane-bound lytic murein transglycosylase B implicated in cell wall remodeling and cell growth (Hunt and Alexson, 2002). *P. halocryophilus* also increases synthesis of peptidoglycans at cold temperatures, and forms a protective envelope on its surface made up partly of peptidoglycan (Mykytczuk et al., 2013, 2015; Raymond-Bouchard et al., 2017).

### Transporters

Overall, numerous transcripts for transporters were strongly increased in both *Rhodococcus* sp. JG3 and *Polaromonas* sp. Eur3 1.2.1 at their colder temperatures compared to the warmer temperatures. In *Rhodococcus* sp. JG3, these were primarily branched-chain amino acid and peptide transporters, while *Polaromonas* sp. Eur3 1.2.1 increased a polar amino acid transport system. Both organisms upregulated sulfate transport and MFS family efflux pumps, including multidrug transporters in *Rhodococcus* sp. JG3, which can recognize a variety of substrates and may also be important for toxin removal. A dicarboxylate symporter was strongly induced in *Rhodococcus* sp. JG3 at -5°C compared to 25°C (>sevenfold; **Supplementary Table S2**), while in *Polaromonas* sp. Eur3 1.2.1 two tripartite tricarboxylate transporter receptor components, *tctC*, and a TRAP-type dicarboxylate transport system were increased two- to fivefold (**Supplementary Table S3**) at 0°C compared to the 20°C condition. Substrates for these transporters can include a variety of carboxylate containing molecules, including malate, fumarate, 2-oxobutyrate, and pyruvate, as well as the compatible solutes ectoine (Mulligan et al., 2011), which may be relevant for osmoregulation (discussed below). The most upregulated (5.6 logFC) transporter transcripts in *Polaromonas* sp. Eur3 1.2.1 at 0°C relative to 20°C were from an *ompA-ompF* type porin, which forms a channel for uptake of a variety of hydrophilic solutes (Confer and Ayalew, 2013). These types of porins were also increased at 4°C in the Antarctic bacterium *Shewanella livingstonensis* (Kawamoto et al., 2007). Overall, increasing transporters at low temperatures is an important strategy for eury- and steno-psychrophiles (Mock et al., 2005; Campanaro et al., 2011; Koh et al., 2017), who must overcome reduced diffusion rates and ensure efficient nutrient uptake (Nedwell, 1999).

### Amino Acid Metabolism

In addition to the large increases in expression of amino acid transporters (discussed above), *Rhodococcus* sp. JG3 strongly upregulated transcripts for two of the enzymes involved in methionine biosynthesis at -5°C compared to 25°C, MetE and MetF, by four- and fivefold, respectfully (**Supplementary Table S2**), likely due to this amino acid’s importance in protein biosynthesis. In addition, methionine is a precursor to the major cellular methyl donor in methylation reactions, *S*-adenosyl methionine (Figge, 2006), and is consistent with increases in several methyltransferases during growth at -5°C (**Supplementary Tables S2, S4**). Smaller increases (<threefold) in expression of transcripts for enzymes involved in the synthesis of glutamine, cysteine, and branched-chain amino acids were also observed. Increases in amino acid synthesis was an important part of the response to cold growth in the marine bacterium *Sphingopyxis alaskensis* (Ting et al., 2010). Also of note is the induced presence of transcripts for two enzymes involved in the Kynurenine pathway, KynA and KynU, leading to the production of the co-enzyme nicotinamide adenine dinucleotide (NAD+) from the catabolism of tryptophan. NAD+ is an important co-factor in numerous cellular processes.

### Carbon, Energy, and Co-enzyme Metabolism

Transcripts for numerous enzymes involved in glycolysis, D-mannose metabolism, the electron transport chain (ETC) and oxidative phosphorylation were increased in *Polaromonas* sp. Eur3 1.2.1 at 0°C compared to 20°C, including all subunits of NADH-quinone oxidoreductase, ATP synthase, cytochrome c553, triosephosphate isomerase, and mannose-6-phosphate isomerase (**Supplementary Table S3**). The increased abundance of all subunits of tetrathionate reductase highlights the potential for *Polaromonas* sp. Eur3 1.2.1 to utilize tetrathionate as a terminal electron acceptor and may be an important strategy for growth in permafrost at subzero temperatures by providing respiratory flexibility, including anaerobic respiration, for energy generation. The ability of stenopsychrophiles to have higher growth rates at cold temperatures has been attributed, at least in part, to the ability to increase energy acquisition. Photosynthesis in the stenopsychrophiles *Fragilariopsis cylindrus* and *Chlorella* sp. UMACC 234 was increased at -1 and 4°C, respectively (Mock and Hoch, 2005; Mock et al., 2005; Chong et al., 2011). The increase in expression of genes involved in oxidative phosphorylation may explain how *Polaromonas* sp. Eur3 1.2.1 is able to maintain growth rates at 0°C (0.19 d^-1^) that are very similar to >20°C (.21 d^-1^), by keeping energy metabolism at a constant rate and offsetting reduced reaction rates at low temperatures.

These same increases were not seen in *Rhodococcus* sp. JG3 for ETC and glycolysis, and in fact multiple cytochromes were decreased at -5°C compared to 25°C (**Supplementary Tables S2, S4**). However, there was a sevenfold upregulation of L-lactate dehydrogenase, responsible for either the conversion of L-lactate to pyruvate or the reverse reaction. There was also significant upregulation of genes involved in co-factor biosynthesis, biotin, NAD, porphyrin, and Fe–S clusters, and for maintaining redox potential. Amongst those most induced was six- and sevenfold upregulation of two enzymes for protoporphyrinogen IX oxidase, menaquinone-dependent, required for porphyrin synthesis (**Supplementary Table S2**). A pimeloyl-ACP methyl ester carboxylesterase, almost fivefold upregulated, functions as a gatekeeper to remove the methyl group from pimeloyl-ACP methyl ester producing pimeloyl-ACP which stops further elongation of fatty acid synthesis and shuttles pimeloyl-ACP into the biosynthesis pathway for the co-factor biotin (Agarwal et al., 2012). Iron–sulfur cluster biosynthesis was also increased (discussed above). Maintenance of redox potential could be observed at -5°C in *Rhodococcus* sp. JG3 through the increased presence of numerous dehydrogenases and oxidoreductases (**Supplementary Tables S2, S4**) when compared to 25°C. Overall, these results indicate that *Rhodococcus* sp. JG3 places emphasis on preserving cellular activity through redox homeostasis and co-factor synthesis at subzero temperatures.

The ability of *Rhodococcus* sp. JG3 to catabolize alcohols under subzero conditions was observed with large fold-change increases in homologs of a propanol-preferring alcohol dehydrogenase (7.21 logFC) and a secondary alcohol dehydrogenase (5.5 logFC). The large subunit of ethanolamine ammonia-lyase (*eutB*) was increased 6.5-fold, and is responsible, along with smaller subunit *eutC*, for cleaving ethanolamine to ammonia and ethanol, and subsequently, acetaldehyde (Tsoy et al., 2009). This suggests the potential for secondary alcohols, aromatic alcohols, and ethanolamine to serve as carbon sources and, in the case of ethanolamine, a nitrogen source as well (Roof and Roth, 1988). Numerous members of the genus *Rhodococcus* are capable of breaking down secondary and aromatic alcohols (Ludwig et al., 1995; Peng et al., 2006).

Finally, while strongly suppressed at -5°C relative to 25°C, all of the genes involved in the four steps of the phenylacetic acid catabolism pathway for the breakdown of aromatic compounds (*paaA*, *paaB*, *paaC*, *paaD*, *paaE*, *paaG*, *paaI*, *paaZ*, and *paaH*) were expressed in *Rhodococcus* sp. (**Supplementary Table S2**). While this pathway does not appear to be important for growth of *Rhodococcus* sp. at -5°C, where it may be preferable to focus available energy on streamlining primary metabolism and downregulate secondary metabolic processes, it is nevertheless worthwhile to note the catabolic potential of this organism.

### Oxidative and Universal Stress Responses

There is some evidence that oxidative stress is higher at colder temperatures due to increased gas solubility and increases in rates of enzyme activity to adapt to reduced catalytic rates at lower temperatures (Chattopadhyay et al., 2011; De Maayer et al., 2014). This appears to be the case in *Rhodococcus* sp. JG3 and *Polaromonas* sp. Eur3 1.2.1 given the increase in transcripts associated with iron acquisition and redox potential observed at -5°C compared to 25°C in *Rhodococcus* sp. JG3, and the emphasis on formation and activity of Fe–S cofactors by both *Rhodococcus* sp. JG3 and *Polaromonas* sp. Eur3 1.2.1 at their respective colder temperatures when compared to their respective higher temperatures. Iron is involved in the production of oxygen radicals *via* the Fenton reaction (Fenton, 1894; Troxell and Hassan, 2013). To relieve oxidative stress, two catalases and a cytochrome peroxidase were increased almost twofold in *Polaromonas* sp. Eur3 1.2.1 (**Supplementary Table S3**). In *Rhodococcus* sp. JG3, a specific catalase gene was upregulated ∼threefold, replacing three other catalase genes, which were downregulated (**Supplementary Table S2**). A superoxide dismutase, responsible for converting the superoxide radical to oxygen or hydrogen peroxide, was also increased in *Rhodococcus* sp.

*Rhodococcus* sp. and *Polaromonas* sp. Eur3 1.2.1 possess 16 and 13 copies, respectively, of the uspA in their genome (Goordial et al., 2016; Raymond-Bouchard et al., 2018). Four of these were strongly (3.5- to 6.5-fold) induced during subzero growth in *Rhodococcus* sp. JG3 (**Supplementary Table S2**) compared to 25°C, while one copy of was upregulated about 3.5-fold in *Polaromonas* sp. Eur3 1.2.1, and two copies were decreased (**Supplementary Table S3**) from the higher to lower temperature. While the exact function of this family of genes remains poorly elucidated, they are induced by a variety of environmental stressors, such as nutrient starvation, extreme temperatures, high salinity, and drought. They are thought to protect the cell from stress and damage and in some cases are linked to protection against DNA-damaging agents (Kvint et al., 2003). Further studies looking into the function of uspA genes at low temperatures in *Rhodococcus* sp. JG3 and *Polaromonas* sp. Eur3 1.2.1 would be interesting, given their selective upregulation and differential expression patterns.

### Compatible Solutes and Osmoregulation

Compatible solutes, small water soluble organic compounds, are accumulated by cold-adapted microbes and play an important role in resisting osmotic pressure caused by high salinity and low water activity associated with cryoenvironments, such as the brine veins in permafrost and ice (Doyle et al., 2012). In addition, compatible solutes also play a role as cryoprotectants and increase the stability of macromolecules, membranes, and proteins, as well as enhancing folding and ligand binding in the latter (Thomas et al., 2001; Yancey, 2005). Genes for the biosynthesis or transport of compatible solutes are induced at cold temperatures in several cold-adapted strains, including *E. sibiricum*, *M. burtonii*, and *P. arcticus* (Rodrigues et al., 2008; Bergholz et al., 2009; Campanaro et al., 2011), as well as this study. The choline transporter *betT* was increased by threefold, and components of the *opuABCD* system for uptake of the compatible solute glycine betaine were over fivefold increased at -5°C in *Rhodococcus* sp. JG3, as was a putative osmotically induced protein (*osmC*) (**Supplementary Table S2**) when compared to 25°C. OsmC in *Mycobacterium* spp. functions as a hydroperoxide reductase and may therefore have a role as an antioxidant in *Rhodococcus* sp. JG3 (Saikolappan et al., 2011). However, *betT* was increased, and genes involved in the synthesis of glycine betaine, *betA* and *betB*, were more than six- and eightfold decreased in *Rhodococcus* sp. JG3. This might indicate preference for the uptake of available solutes from the environment, rather than energy-demanding *de novo* synthesis of solutes. It is also possible that the synthesis genes were induced during the initial acclimation period and were subsequently downregulated once optimal concentrations of compatible solutes had been achieved, and thus were not detected in these cultures which were harvested at mid-late exponential phase. A similar theory has been proposed for *P. halocryophilus* (Mykytczuk et al., 2013).

*Polaromonas* sp. Eur3 1.2.1 does not appear to upregulate transport of compatible solutes to the extent that *Rhodococcus* sp. JG3 does, though this may be explained by the fact that the media used to culture *Polaromonas* sp. Eur3 1.2.1 did not contain additional NaCl beyond what was present in the growth medium (R2A). Nevertheless, *Polaromonas* sp. Eur3 1.2.1 also increases the abundance of transcripts for an osmotically inducible protein (*osmB*) by almost fourfold (**Supplementary Table S3**). A >threefold increase in a TRAP system capable of transporting ectoine suggests the potential for accumulation of compatible solutes. Even under low salt conditions, cold growth appears to be linked at least partially with osmoregulation systems.

### Transcription, Signaling, and Motility

Numerous transcription factors (TFs) were strongly upregulated during cold-temperature growth in *Rhodoccocus* sp. and in *Polaromonas* sp. Eur3 1.2.1, though slightly less so. The predicted regulatory functions of these TFs are consistent with many transcripts increased in abundance in both organisms. Ten transcriptional regulators were found to be >threefold upregulated in *Rhodoccocus* sp. at -5°C compared to 25°C, with 9 more than fivefold upregulated (**Supplementary Tables S2, S4**), including two *acrR* family regulators, known to be involved in modulating responses to osmotic stress, modification and elimination of toxic substances, and lipid metabolism (Deng et al., 2013), the primary sigma-70 factor required for transcription initiation (Paget and Helmann, 2003), and the *whiB7* transcriptional regulator, which is highly conserved in actinomycetes, with characterized roles in redox homeostasis, cell metabolism, and antibiotic resistance (Burian et al., 2012).

NsrR is a nitric oxide (NO) sensitive transcription repressor which can upregulate the ResDE two-component system required for induction of nitrate respiration genes and NO detoxifying enzymes in the presence of NO (Yukl et al., 2008). The elevated transcription of *nsrR* and the flavohemoglobin NO-detoxifying enzyme *hmp* suggests that *Rhodococcus* sp. JG3 may be under heightened NO stress at subzero temperatures when compared to higher temperatures. One of the genes involved in nitrate reduction, nitrite reductase, was detected, suggesting that *Rhodococcus* sp. JG3 may be capable of denitrification but it was >4.5-fold downregulated at -5°C relative to 25°C. MarR (∼sixfold increase) is important in the stress response and modification and export of toxic compounds, partly through the induction of efflux pumps (Grove, 2013), and *ytrA* (5.5-fold increased) is involved in regulation of ATP-binding cassette transporters (Suvorova et al., 2015). These TFs may have roles in the induction of the MFS pumps and ABC transporters observed in *Rhodococcus* sp. JG3 at -5°C (discussed above).

At cold temperatures both *Rhodococcus* sp. JG3 and *Polaromonas* sp. Eur3 1.2.1 show increased levels of transcription factors of the *narL/fixJ/luxR* and *iclR* families (**Supplementary Tables S2, S3**). In addition, *Polaromonas* sp. Eur3 1.2.1 induces 2 *lysR* family TFs and a regulator of the *gntR* family, the same family as ytrA in *Rhodococcus* sp. JG3 during 0°C growth as compared to 20°C. Members of the *iclR* family are transcriptional activators and repressors often involved in regulating carbon metabolism, such as the glyoxylate bypass operon, degradation of aromatic compounds, quorum-sensing, and multidrug resistance (Sunnarborg et al., 1990; Chao and Zhou, 2013). *narL/fixJ/luxR* comprises a large family of TFs and, therefore, the exact role of these in *Polaromonas* sp. Eur3 1.2.1 and *Rhodococcus* sp. JG3 is hard to pinpoint but potential roles could include activating the nitrate reductase operon and nitrogen-fixation genes and uptake of the compatible solute ectoine (Rabin and Stewart, 1993; Galiniers et al., 1994; Rodríguez-Moya et al., 2010). The presence of the *nac/lysR* regulator, or nitrogen assimilation control protein, in *Polaromonas* sp. Eur3 1.2.1 would suggest that at cold temperatures *Polaromonas* sp. Eur3 1.2.1 experiences nitrogen limitation (Bender, 2010). Signaling systems associated with these transcription factors were induced about two- to threefold at 0°C in *Polaromonas* sp. Eur3 1.2.1 (**Supplementary Tables S3, S5**) including the two-component system of *fixJ/luxR*, adenylate cyclase, and components of the cAMP receptor protein, a global transcriptional activator that regulates transcription of many genes, including energy metabolism, consistent with increases in energy metabolism observed in the bacterium at 0°C. Overall, the transcription factors upregulated at cold temperatures suggest the induction of numerous pathways important during cold growth, including lipid metabolism, redox homeostasis, ABC transporters, and osmoregulation in *Rhodococcus* sp. JG3, and ABC transporters, carbon and energy metabolism, and export of toxic compounds in *Polaromonas* sp. Eur3 1.2.1.

## Conclusion

**FIGURE 2:**
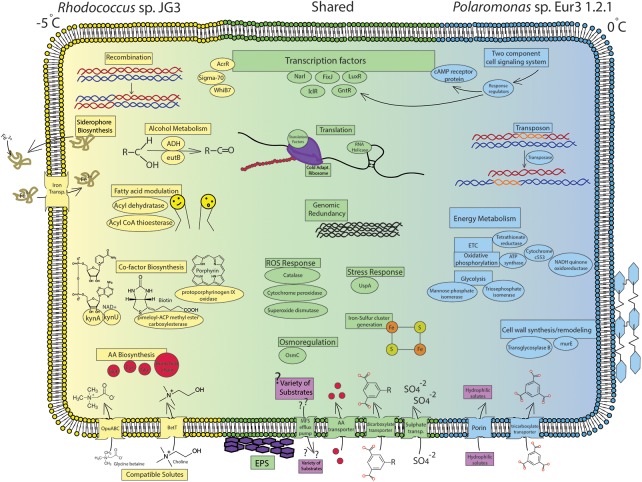
Cell diagram showing important processses and pathways increased during cold growth. Blue labels represent processes increased in *Polamonas* sp. Eur3 1.2.1 at 0°C, yellow labels represent processes increased in *Rhodococcus* sp. JG3 at –5°C, and green labels represent shared processes increased in both organisms during cold growth. Full gene descriptions corresponding to the short protein names used in the figure can be found in **Supplementary Tables S2–S5**.

Overall, the transcriptomic responses to cold growth in *Rhodococcus* sp. and *Polaromonas* sp. Eur3 1.2.1, shared many cold-adaptive features (**Figure 2**). Common responses included induction of translation and ribosomal processes, which we hypothesize results in translationally active cold-adapted ribosomes, upregulation of nutrient transport, increased oxidative and osmotic stress responses, modulating cell wall/membrane features, induction of EPS synthesis, and accumulation of compatible solutes, though this last item was much more pronounced in *Rhodococcus* sp. Recombination and genomic redundancy also appeared to be a shared strategy at low temperatures, though the mechanism used to achieve this was different in each organism. *Polaromonas* sp. Eur3 1.2.1 utilized specific transposases, while *Rhodococcus* sp. induced recombination proteins. The presence of the above properties in most psychrophiles, whether eury- or steno-, suggests that these may be conserved adaptive features, necessary for growth at low temperatures in most organisms.

In *Rhodococcus* sp. JG3, the marked differences during cold growth featured increased abundance of transcripts involved in iron transport, amino acid transport and metabolism, modulating fatty acid synthesis and composition, catabolism of alcohols/ethanolamine, and sustaining redox potential (**Figure 2**). Conversely, *Polaromonas* sp. Eur3 1.2.1 was found to induce energy metabolism relating to the ETC, oxidative phosphorylation, and glycolysis, as well as global signal transduction mechanisms and transport and metabolism of carboxylates (**Figure 2**). In addition, enzymes vital to peptidoglycan synthesis and modulation were increased. Finally, *Polaromonas* sp. Eur3 1.2.1 differentially up/downregulated a specific subset of transposases at the two growth temperatures.

As mentioned previously, the two organisms used in this study are from two different phyla. As such, we were able to investigate relative changes that may be largely conserved cold adaptations vs. ones that may be specific to certain groups. Though we have yet to identify such candidates from permafrost, in the future, comparing a steno-and a eury-psychrophile from the same genus would also be worthwhile to narrow down more specific adaptive features. It is also important to note that some functions involved in cold adaptation may not be regulated at the transcriptional level and it is likely that some regulation occurs at the posttranscriptional level as well. Overall, however, this study does provide us with valuable insight into the cold-adaptive strategies of two different cold-adapted organisms. We also recognize that we focused exclusively on RNA-seq data. Our goal was to identify genes and mechanisms of interest that may be important during cold growth to provide a basis for future studies.

Increased activity of primary energy production and the ETC in *Polaromonas* sp. Eur3 1.2.1 and other stenopsychrophiles, is likely to be one major mechanism by which these organisms are able to sustain optimal growth at colder temperatures or consistent growth rates over their temperature range. The more attenuated transcriptomic response in *Polaromonas* sp. Eur3 1.2.1 also suggests that the organism may be, at least partly, constitutively adapted to colder temperatures or for growth over its temperature range and does not need to upregulate its response to the same levels as *Rhodococcus* sp. JG3 at lower temperatures. However, more research will need to be done to determine if this is true and to identify those features which are unique to these types of organisms, and differ from eurypsychrophiles. Certainly, it may simply be that stenopsychrophiles are adapted to grow at a narrow range and temperature changes, both above and below this range, are difficult for these organisms. In the same vein, it is difficult to pinpoint the mechanisms that favor eurypsychrophilic growth at subzero temperatures from this study alone, though in the case of *Rhodococcus* sp. JG3 increased co-factor formation, flexibility in use of carbon sources, control of fatty acid composition in the membrane, iron acquisition, and sustaining redox potential appear to be important factors. These may represent adaptive strategies that allow this organism to grow at subzero temperatures, in addition to sustaining growth over a wide range of temperatures (-5 to 30°C). It is hoped that further studies will better elucidate the role that these mechanisms may play in cold growth in both eury- and steno-psychrophiles.

## Author Contributions

IR-B wrote the manuscript, performed all laboratory experiments, and all downstream analyses with the results of the pipeline data. JT performed bioinformatic analysis of the raw RNAseq data using the transcriptomic pipeline, provided experimental guidance, and manuscript feedback and editing. IA helped with creation of the cell figure, assisted with library preparation, and participated in manuscript editing and discussion. CG and LW provided guidance, and critical feedback and editing of the manuscript.

## Conflict of Interest Statement

The authors declare that the research was conducted in the absence of any commercial or financial relationships that could be construed as a potential conflict of interest.

## Footnotes

1 http://bio-bwa.sourceforge.net

2 https://img.jgi.doe.gov/cgi-bin/mer/main.cgi

3 https://www.bioconductor.org/packages/3.3/bioc/vignettes/edgeR/inst/doc/edgeRUsersGuide.pdf

4 http://www.ncbi.nlm.nih.gov/bioproject/422596

5 https://img.jgi.doe.gov/cgi-bin/mer/main.cgi
