# Supplementary material for: Comparative Transcriptomics of Cold Growth and Adaptive Features of a Eury- and Steno-Psychrophile
Source: Front Microbiol. 2018 Jul 31;9:1565. doi: 10.3389/fmicb.2018.01565 (PMC6080646; doi:10.3389/fmicb.2018.01565)
Supplement: Supplementary file 1 [file Table_1.docx]

***Supplementary Material***

**Comparative Transcriptomics of Cold Growth and Adaptive Features of a Eury- and Steno-psychrophile**

**Isabelle Raymond-Bouchard^1^, Julien Tremblay^2^, Ianina Altshuler^1^, Charles Greer^2^, Lyle G. Whyte^1*^**

***Correspondence**: Lyle Whyte: [lyle.whyte@mcgill.ca](mailto:lyle.whyte@mail.mcgill.ca)

**Supplementary Table 1**

**Table S1**. Sequencing summary for all samples used in this study. Letters in the sample name (A, B, C, and D) represent a specific growth condition, each sampled in triplicate. A = *Polaromonas* sp Eur3 1.2.1 at 20°C; B = *Polaromonas* sp Eur3 1.2.1 at 0°C; C = *Rhodococcus* sp. JG3 at 25°C; D = *Rhodococcus* sp. JG3 at -5°C; RIN = RNA integrity number

| Name | Sample Name | RIN | Raw Fragments | Surviving Fragments | Surviving Fragments% | Surviving Single | Total Reads QCed | mapped | mapped% | Properly Paired | Properly Paired% |
| --- | --- | --- | --- | --- | --- | --- | --- | --- | --- | --- | --- |
| Pola | A1_S1_L001 | 9.4 | 558,396 | 540,271 | 96% | 119 | 1,077,884 | 441,110 | 40% | 427,206 | 39% |
| Pola | A2_S2_L001 | 9 | 333,878 | 330,776 | 99% | 163 | 659,318 | 279,583 | 42% | 266,218 | 40% |
| Pola | A3_S3_L001 | N/A | 1,570,954 | 1,553,501 | 98% | 275 | 3,071,830 | 1,274,738 | 41% | 1,219,650 | 39% |
| Pola | B4_S4_L001 | 9.3 | 316,915 | 315,531 | 99% | 46 | 628,998 | 313,978 | 49% | 297,224 | 47% |
| Pola | B5_S5_L001 | 8.7 | 273,550 | 270,447 | 98% | 56 | 538,694 | 281,568 | 52% | 262,892 | 48% |
| Pola | B7_S6_L001 | 9.7 | 630,322 | 625,343 | 99% | 55 | 1,241,862 | 540,485 | 43% | 508,496 | 40% |
| Rhodo | C1_S7_L001 | 8.8 | 157,968 | 129,930 | 82% | 26 | 255,956 | 173,352 | 67% | 170,482 | 66% |
| Rhodo | C2_S8_L001 | 8.4 | 292,608 | 272,733 | 93% | 65 | 541,856 | 407,084 | 75% | 402,236 | 74% |
| Rhodo | C3_S9_L001 | 8.1 | 195,126 | 193,145 | 98% | 16 | 384,022 | 291,804 | 75% | 286,452 | 74% |
| Rhodo | D1_S10_L001 | 3.8 | 267,891 | 259,603 | 96% | 78 | 512,126 | 345,896 | 67% | 340,234 | 66% |
| Rhodo | D2_S11_L001 | N/A | 128,679 | 126,765 | 98% | 15 | 251,742 | 164,110 | 65% | 159,418 | 63% |
| Rhodo | D3_S12_L001 | N/A | 126,551 | 125,573 | 99% | 14 | 249,964 | 179,168 | 71% | 171,820 | 68% |
|  |  |  | 4,852,838 | 4,743,618 |  | 928 | 9,414,252 | 4,692,876 |  | 4,512,328 |  |
| raw bp | 727.93 |  |  |  |  |  |  |  |  |  |  |
