# Supplementary material for: Comparative Transcriptomics of Cold Growth and Adaptive Features of a Eury- and Steno-Psychrophile
Source: Front Microbiol. 2018 Jul 31;9:1565. doi: 10.3389/fmicb.2018.01565 (PMC6080646; doi:10.3389/fmicb.2018.01565)
Supplement: Supplementary file 2 [file Table_2.docx]

***Supplementary Material***

**Comparative Transcriptomics of Cold Growth and Adaptive Features of a Eury- and Steno-psychrophile**

**Isabelle Raymond-Bouchard^1^, Julien Tremblay^2^, Ianina Altshuler^1^, Charles Greer^2^, Lyle G. Whyte^1*^**

***Correspondence**: Lyle Whyte: [lyle.whyte@mcgill.ca](mailto:lyle.whyte@mail.mcgill.ca)

**Supplementary Table 2**

**Table S2.** Select genes with the highest differential expression at -5°C compared to 25°C in *Rhodococcus* sp. JG3. logFC is log fold change at -5°C as compared to 25°C. Includes COG and KEGG Orthology (KO) assignments.

| **Gene** | **Gene Description** | **Shortname** | **logFC** | **COG** | **KO** |
| --- | --- | --- | --- | --- | --- |
| ***Amino acid metabolism*** | |  |  |  |  |
| 2529301904 | 5,10-methylenetetrahydrofolate reductase | metF | 5.31 | COG0685 | K00297 |
| 2529300492 | Methionine synthase II (cobalamin-independent) | metE | 4.08 | COG0620 | K00549 |
| ***Cell wall/membrane and EPS biosynthesis*** | |  |  |  |  |
| 2529303100 | Heparin binding hemagglutinin | hbhA | 6.13 |  | K16645 |
| 2529302855 | Acyl-ACP thioesterase |  | 5.94 | COG3884 |  |
| 2529302771 | Exopolysaccharide biosynthesis protein, predicted pyruvyl transferase | epsO | 3.15 | COG5039 | K19431 |
| 2529299791 | Sugar transferase involved in LPS biosynthesis (colanic, teichoic acid) |  | 3.02 | COG2148 |  |
| 2529302388 | Acyl dehydratase |  | 2.30 | COG2030 |  |
| 2529302869 | Putative flippase GtrA | gtrA | 2.21 | COG2246 |  |
| ***Iron transport and acquisition*** | |  |  |  |  |
| 2529300144 | NADPH-dependent ferric siderophore reductase, contains FAD-bin |  | 8.13 | COG2375 |  |
| 2529300140 | Mycobactin salicyl-AMP ligase | mbtA | 6.33 | COG1021 | K04787 |
| 2529301663 | ABC-type cobalamin/Fe3+-siderophores transport system, ATPase | ABC.FEV.A | 6.17 | COG1120 | K02013 |
| 2529300145 | Salicylate synthetase | mbtI | 6.02 | COG0147 | K04781 |
| 2529302627 | ABC-type Fe3+-hydroxamate transport system, periplasmic component | ABC.FEV.S | 5.70 | COG0614 | K02016 |
| 2529301661 | ABC-type Fe3+-siderophore transport system, permease component | ABC.FEV.P | 5.35 | COG0609 | K02015 |
| 2529300143 | ABC-type Fe3+-hydroxamate transport system, periplasmic component | ABC.FEV.S | 5.21 | COG0614 | K02016 |
| 2529303132 | ABC-type Fe3+-hydroxamate transport system, periplasmic component | ABC.FEV.S | 4.71 | COG0614 | K02016 |
| 2529300593 | Fe2+ uptake regulator, Fur family transcriptional regulator | fur | 4.49 | COG0735 | K03711 |
| 2529300147 | L-ornithine N5-oxygenase | pvdA | 4.46 | COG3486 | K10531 |
| 2529300139 | Mycobactin phenyloxazoline synthetase | mbtB | 3.51 | COG1020 | K04788 |
| 2529301174 | NADPH-dependent ferric siderophore reductase, contains FAD-bin |  | 2.95 | COG2375 |  |
| 2529299468 | ABC-type Fe3+-hydroxamate transport system, periplasmic component | ABC.FEV.S | 2.32 | COG0614 | K02016 |
| 2529303362 | ABC-type Fe3+ transport system, periplasmic component | afuA, fbpA | 2.26 | COG1840 | K02012 |
| 2529300022 | Fe2+ uptake regulator, Fur family transcriptional regulator | fur | -4.40 | COG0735 | K03711 |
| ***Transporters*** | |  |  |  |  |
| 2529299875 | ABC-type multidrug transport system, ATPase component | ABC-2.A | 7.56 | COG1131 | K01990 |
| 2529302312 | Na+/H+-dicarboxylate symporter |  | 7.21 | COG1301 |  |
| 2529302083 | ABC-type sulfate transport system, permease component | cysW | 5.93 | COG4208 | K02047 |
| 2529302491 | ABC-type branched-chain amino acid transport system, permease | livM | 5.69 | COG4177 | K01998 |
| 2529300854 | ABC-type sugar transport system, periplasmic component | ABC.SS.S | 5.18 | COG1879 | K02058 |
| 2529302471 | ABC-type dipeptide/oligopeptide/nickel transport system, permease | ABC.PE.P | 5.16 | COG0601 | K02033 |
| 2529301448 | ABC-type peptide/nickel transport system, periplasmic component | ABC.PE.S | 3.54 | COG0747 | K02035 |
| 2529300505 | Aerobic C4-dicarboxylate transport protein | dctA | 3.09 | COG1301 | K11103 |
| 2529301861 | MFS transporter, DHA2 family, multidrug resistance protein | smvA, qacA, lfrA | 3.01 |  | K08167 |
| 2529301796 | ABC-type branched-chain amino acid transport system, periplasmic comp | livK | 2.35 | COG0683 | K01999 |
| 2529300180 | ABC-type peptide/nickel transport system, periplasmic component | ABC.PE.S | 2.29 | COG0747 | K02035 |
| 2529301795 | ABC-type branched-chain amino acid transport system, ATPase | livF | 1.66 | COG0410 | K01996 |
| 2529301792 | Branched-chain amino acid ABC-type transport system, permease | livH | 1.59 | COG0559 | K01997 |
| 2529299546 | ABC-type dipeptide/oligopeptide/nickel transport system, permease | ABC.PE.P | -4.57 | COG0601 | K02033 |
| ***Carbon, Energy, and co-enzyme metabolism*** | |  |  |  |  |
| 2529299871 | Protoporphyrinogen IX oxidase, menaquinone-dependent (flavodoxin) | hemG | 7.62 | COG4635 | K00230 |
| 2529299865 | Protoporphyrinogen IX oxidase, menaquinone-dependent (flavodoxin) | hemG | 6.73 | COG4635 | K00230 |
| 2529300710 | Ethanolamine ammonia-lyase, large subunit | eutB | 6.49 | COG4303 | K03735 |
| 2529299868 | Alcohol dehydrogenase, propanol-preferring | adhP | 7.21 | COG1064 | K13953 |
| 2529300697 | Glycolate oxidase/L-lactate dehydrogenase (cytochrome) | glcD, lldD | 7.01 | COG1304 | K00104 |
| 2529300499 | Nitric oxide dioxygenase | hmp, YHB1 | 5.96 | COG1017 | K05916 |
| 2529300276 | Pimeloyl-ACP methyl ester carboxylesterase |  | 4.77 | COG0596 |  |
| 2529303159 | NAD+-dependent secondary alcohol dehydrogenase | adh1 | 5.50 | COG1064 | K18382 |
| 2529298821 | 2-(1,2-epoxy-1,2-dihydrophenyl) acetyl-CoA isomerase | paaG | -6.73 | COG1024 | K15866 |
| 2529298822 | ring 1,2-phenylacetyl-CoA epoxidase, catalytic subunit PaaA | paaA | -5.37 | COG3396 | K02609 |
| 2529300992 | NAD(P)H-nitrite reductase, large subunit | nirB | -4.57 | COG1251 | K00362 |
| 2529298827 | Phenylacetate-coenzyme A ligase, adenylate-forming domain | paaK | -4.36 | COG1541 | K01912 |
| 2529298820 | 3-hydroxybutyryl-CoA dehydrogenase | paaH, hbd, fadB | -3.89 | COG1250 | K00074 |
| 2529298826 | ring-1,2-phenylacetyl-CoA epoxidase subunit PaaE | paaE | -3.70 | COG1018 | K02613 |
| 2529298823 | ring 1,2-phenylacetyl-CoA epoxidase, PaaB subunit | paaB | -3.47 | COG3460 | K02610 |
| 2529298825 | ring-1,2-phenylacetyl-CoA epoxidase subunit PaaD | paaD | -3.22 | COG2151 | K02612 |
| 2529298824 | 1,2-phenylacetyl-CoA epoxidase, catalytic subunit | paaC | -2.38 | COG3396 | K02611 |
| 2529298814 | Acyl-coenzyme A thioesterase PaaI, contains HGG motif | paaI | -2.15 | COG2050 | K02614 |
| ***Stress and oxidative responses*** | |  |  |  |  |
| 2529299876 | Nucleotide-binding universal stress protein, UspA family |  | 6.53 | COG0589 |  |
| 2529299882 | Nucleotide-binding universal stress protein, UspA family |  | 6.26 | COG0589 |  |
| 2529299879 | Nucleotide-binding universal stress protein, UspA family |  | 4.44 | COG0589 |  |
| 2529299880 | Nucleotide-binding universal stress protein, UspA family |  | 3.55 | COG0589 |  |
| 2529300594 | Catalase (peroxidase I) | katG | 3.17 | COG0376 | K03782 |
| 2529299042 | Superoxide dismutase | SOD2 | 1.53 | COG0605 | K04564 |
| 2529300023 | Catalase (peroxidase I) | katG | -5.95 | COG0376 | K03782 |
| 2529300031 | Mn-containing catalase (includes spore coat protein CotJC) |  | -5.10 | COG3546 | K07217 |
| 2529299729 | Catalase | katE, CAT, catB | -3.64 | COG0753 | K03781 |
| ***Translation, ribosomes, helicases, posttranslational modifications*** | |  |  |  |  |
| 2529302299 | ADP-ribosylglycohydrolase |  | 5.16 | COG1397 |  |
| 2529300328 | Superfamily II DNA and RNA helicase |  | 3.75 | COG0513 |  |
| 2529298761 | Queuine/archaeosine tRNA-ribosyltransferase | tgt | 3.45 | COG0343 | K00773 |
| 2529301229 | Ribosome-binding factor A | rbfA | 3.45 | COG0858 | K02834 |
| 2529301134 | Ribosomal protein L28 |  | 3.18 | COG0227 | K02902 |
| 2529302298 | O-acetyl-ADP-ribose deacetylase (regulator of RNase III) |  | 3.12 | COG2110 |  |
| 2529299072 | Regulator of RNase E activity RraA | rraA, menG | 2.79 | COG0684 | K02553 |
| 2529301894 | 16S rRNA C1402 N4-methylase RsmH | mraW, rsmH | 2.77 | COG0275 | K03438 |
| 2529300757 | Ribosomal protein S5 | RP-S5, rpsE | 2.70 | COG0098 | K02988 |
| 2529300756 | Ribosomal protein L18 | RP-L18, rplR | 2.25 | COG0256 | K02881 |
| 2529301143 | Ribosomal protein L32 | RP-L32, rpmF | 2.07 | COG0333 | K02911 |
| 2529302902 | Ribosomal protein L7/L12 | RP-L7, rplL | 2.04 | COG0222 | K02935 |
| 2529300742 | Ribosomal protein L29 | RP-L29, rpmC | 2.00 | COG0255 | K02904 |
| 2529302067 | GTPase Era, involved in 16S rRNA processing | era | 2.33 | COG1159 | K03595 |
| 2529300958 | Ribosome-associated translation inhibitor RaiA | raiA | -2.18 | COG1544 |  |
| 2529301311 | mRNA degradation ribonuclease J1/J2 | rnj | -1.61 | COG0595 | K12574 |
| ***DNA recombination*** | |  |  |  |  |
| 2529299297 | Single-strand DNA-binding protein | ssb | 2.44 | COG0629 | K03111 |
| 2529301443 | Holliday junction DNA helicase RuvA | ruvA | 2.01 | COG0632 | K03550 |
| 2529302293 | Exodeoxyribonuclease V alpha subunit | recD | 1.99 | COG0507 | K03581 |
| ***Osmoregulation*** | |  |  |  |  |
| 2529301958 | ABC-type proline/glycine betaine transport system, ATPase component | opuA | 5.49 | COG1125 | K05847 |
| 2529301959 | ABC-type proline/glycine betaine transport system, permease component | opuBD | 5.20 | COG1174 | K05846 |
| 2529302730 | Uncharacterized OsmC-related protein |  | 5.10 | COG1765 |  |
| 2529302675 | Periplasmic glycine betaine/choline-binding (lipo)protein of a ABC-type glycine betaine transport system | opuC | 3.37 | COG1732 | K05845 |
| 2529303167 | Choline-glycine betaine transporter | betT, betS | 3.21 | COG1292 | K02168 |
| 2529298829 | Choline dehydrogenase | betA, CHDH | -8.14 | COG2303 | K00108 |
| 2529298830 | Betaine-aldehyde dehydrogenase | betB, gbsA | -6.46 | COG1012 | K00130 |
| ***Transcription*** | |  |  |  |  |
| 2529301008 | WhiB family transcriptional regulator, redox-sensing regulator | whiB7 | 5.91 |  | K18958 |
| 2529299065 | DNA-binding transcriptional regulator, MarR family |  | 5.73 | COG1846 |  |
| 2529301848 | DNA-binding transcriptional regulator YtrA, GntR family | ytrA | 5.57 | COG1725 | K07979 |
| 2529302494 | DNA-binding transcriptional regulator, IclR family |  | 5.60 | COG1414 |  |
| 2529303412 | RNA polymerase sigma-70 factor, ECF subfamily | SIG3.2, rpoE | 5.51 | COG1595 | K03088 |
| 2529300498 | DNA-binding transcriptional regulator, NsrR | nsrR | 5.34 | COG1959 | K13771 |
| 2529301247 | DNA-binding transcriptional regulator, AcrR family |  | 5.21 | COG1309 |  |
| 2529300684 | DNA-binding response regulator, NarL/FixJ family, contains REC |  | 5.04 | COG2197 |  |
| 2529299715 | DNA-binding transcriptional regulator, AcrR family |  | 3.24 | COG1309 |  |
