# Supplementary material for: Comparative Transcriptomics of Cold Growth and Adaptive Features of a Eury- and Steno-Psychrophile
Source: Front Microbiol. 2018 Jul 31;9:1565. doi: 10.3389/fmicb.2018.01565 (PMC6080646; doi:10.3389/fmicb.2018.01565)
Supplement: Supplementary file 3 [file Table_3.docx]

***Supplementary Material***

**Comparative Transcriptomics of Cold Growth and Adaptive Features of a Eury- and Steno-psychrophile**

**Isabelle Raymond-Bouchard^1^, Julien Tremblay^2^, Ianina Altshuler^1^, Charles Greer^2^, Lyle G. Whyte^1*^**

***Correspondence**: Lyle Whyte: [lyle.whyte@mcgill.ca](mailto:lyle.whyte@mail.mcgill.ca)

**Supplementary Table 3**

**Table S3.** Select genes with the highest differential expression at 0°C compared to 20°C in *Polaromonas* sp. Eur3 1.2.1. logFC is log fold change at 0°C as compared to 20°C. Includes COG and KEGG Orthology (KO) assignments.

| **Gene** | **Gene Description** | **Shortname** | **logFC** | **COG** | **KO** |
| --- | --- | --- | --- | --- | --- |
| ***Cell wall/membrane and EPS biosynthesis*** | |  |  |  |  |
| 2619647124 | OmpA-OmpF porin, OOP family | TC.OOP, ompA | 5.62 | COG2885 | K03286 |
| 2619646989 | Outer membrane lipoprotein SlyB |  | 2.88 | COG3133 |  |
| 2619645993 | Undecaprenyl diphosphate synthase | uppS | 2.31 | COG0020 | K00806 |
| 2619647584 | Uncharacterized protein involved in exopolysaccharide biosynthesis |  | 2.14 | COG3206 |  |
| 2619644456 | Putative colanic acid biosysnthesis UDP-glucose lipid carrier transferase | wcaJ | 1.98 | COG2148 | K03606 |
| 2619647025 | Membrane-bound lytic murein transglycosylase B | mltB | 1.91 | COG2951 | K08305 |
| 2619645090 | UDP-N-acetylmuramyl tripeptide synthase | murE | 1.74 | COG0769 | K01928 |
| 2619645525 | Glycosyltransferase, catalytic subunit of cellulose synthase and poly-beta-1,6-N-acetylglucosamine synthase |  | 1.68 | COG1215 |  |
| ***Transporters*** | |  |  |  |  |
| 2619647128 | Tripartite-type tricarboxylate transporter, receptor component TctC | tctC | 5.16 | COG3181 | K07795 |
| 2619645035 | Magnesium/Calcium-transporting ATPase (P-type) |  | 4.49 | COG0474 | K01537 |
| 2619646220 | Predicted arabinose efflux permease, MFS family |  | 3.59 | COG2814 |  |
| 2619646852 | TRAP-type C4-dicarboxylate transport system, periplasmic component |  | 3.32 | COG1638 |  |
| 2619643636 | ABC-type sulfate transport system, periplasmic component | cysP, sbp | 2.89 | COG1613 | K02048 |
| 2619645167 | Tripartite-type tricarboxylate transporter, receptor component TctC |  | 2.40 | COG3181 |  |
| 2619646850 | TRAP-type C4-dicarboxylate transport system, small permease component |  | 2.32 | COG3090 |  |
| 2619643741 | Polar amino acid transport system ATP-binding protein | ABC.PA.A | 2.11 |  | K02028 |
| 2619645443 | ABC-type polar amino acid transport periplasmic component | ABC.PA.S | 2.10 | COG0834 | K02030 |
| ***Carbon, energy, and co-enzyme metabolism*** | |  |  |  |  |
| 2619646396 | Tetrathionate reductase subunit A | ttrA | 5.69 |  | K08357 |
| 2619646394 | Tetrathionate reductase subunit B | ttrB | 4.60 | COG0437 | K08358 |
| 2619647875 | glucose-6-phosphate 1-epimerase |  | 3.51 | COG0676 | K01792 |
| 2619646395 | Tetrathionate reductase subunit C | ttrC | 2.88 |  | K08359 |
| 2619645020 | Cytochrome c553 |  | 2.56 | COG2863 |  |
| 2619645992 | Mannose-6-phosphate isomerase, cupin superfamily |  | 2.41 | COG0662 |  |
| 2619644322 | Malate/lactate dehydrogenase | mdh | 2.39 | COG0039 | K00024 |
| 2619645995 | Mannose-6-phosphate isomerase, cupin superfamily |  | 2.33 | COG0662 |  |
| 2619646941 | NADH:ubiquinone oxidoreductase subunit 11 or 4L (chain K) | nuoK | 2.07 | COG0713 | K00340 |
| 2619647564 | Fe-S cluster assembly iron-binding protein IscA | iscA | 1.95 | COG0316 | K13628 |
| 2619646526 | Fe-S cluster biogenesis protein NfuA, 4Fe-4S-binding domain |  | 1.66 | COG0694 |  |
| 2619647563 | NifU homolog involved in Fe-S cluster formation | iscU, nifU | 1.57 | COG0822 | K04488 |
| 2619646928 | Triosephosphate isomerase | tpiA | 1.97 | COG0149 | K01803 |
| 2619646935 | NADH:ubiquinone oxidoreductase 24 kD subunit E | nuoE | 1.93 | COG1905 | K00334 |
| 2619644324 | Succinate dehydrogenase/fumarate reductase, cytochrome b subunit | sdhC, frdC | 1.78 | COG2009 | K00241 |
| 2619646939 | Formate hydrogenlyase subunit 6/NADH:ubiquinone oxidoreductase subunit I | nuoI | 1.75 | COG1143 | K00338 |
| 2619646934 | NADH:ubiquinone oxidoreductase subunit D | nuoD | 1.72 | COG0649 | K00333 |
| 2619645466 | FoF1-type ATP synthase, membrane subunit c/Archaeal/vacuolar-type H+-ATPase, subunit K | ATPF0C, atpE | 1.69 | COG0636 | K02110 |
| 2619646938 | NADH:ubiquinone oxidoreductase subunit 1 (H) | nuoH | 1.67 | COG1005 | K00337 |
| 2619646943 | NADH:ubiquinone oxidoreductase subunit 4 (M) | nuoM | 1.67 | COG1008 | K00342 |
| 2619645461 | FoF1-type ATP synthase, beta subunit | ATPF1B, atpD | 1.66 | COG0055 | K02112 |
| 2619645462 | FoF1-type ATP synthase, gamma subunit | ATPF1G, atpG | 1.64 | COG0224 | K02115 |
| 2619646411 | Thiosulfate dehydrogenase | tsdA | 1.56 | COG3258 | K19713 |
| 2619646937 | NADH dehydrogenase/NADH:ubiquinone oxidoreductase subunite G | nuoG | 1.54 | COG1034 | K00336 |
| 2619646933 | NADH:ubiquinone oxidoreductase subunit C | nuoC | 1.53 | COG0852 | K00332 |
| 2619643796 | Glucose-6-phosphate isomerase | GPI, pgi | 1.53 | COG0166 | K01810 |
| ***Signal transduction and motility*** | |  |  |  |  |
| 2619646417 | Two-component response regulator, FixJ family, REC and HTH domains |  | 3.45 | COG4566 |  |
| 2619646293 | cAMP-binding domain of CRP or a regulatory subunit of cAMP-dependent protein kinases |  | 2.95 | COG0664 |  |
| 2619645446 | CBS domain |  | 2.86 | COG0517 |  |
| 2619646418 | Two-component system, LuxR family, sensor histidine kinase TtrS | ttrS | 2.74 |  | K13040 |
| 2619645212 | Two-component system, response regulator | rssB, hnr | 2.57 |  | K02485 |
| 2619647120 | cAMP-binding domain of CRP or a regulatory subunit of cAMP-dependent protein kinases |  | 2.23 | COG0664 |  |
| ***Translation, ribosomes, helicases, chaperones*** | |  |  |  |  |
| 2619646416 | Thiol:disulfide interchange protein DsbG | dsbG | 5.85 | COG1651 | K03805 |
| 2619646415 | Thiol-disulfide isomerase or thioredoxin |  | 4.07 | COG0526 |  |
| 2619646079 | Ribosomal protein S7 | RP-S7, rpsG | 2.51 | COG0049 | K02992 |
| 2619645369 | Ribosomal protein S14 | RP-S14, rpsN | 2.44 | COG0199 | K02954 |
| 2619646414 | Thiol:disulfide interchange protein DsbD | dsbD | 2.74 | COG4232 | K04084 |
| 2619645361 | Translation initiation factor IF-1 | infA | 2.10 | COG0361 | K02518 |
| 2619646692 | Superfamily II DNA and RNA helicase |  | 2.04 | COG0513 |  |
| ***Osmoregulation, stress, and oxidative responses*** | |  |  |  |  |
| 2619646962 | Osmotically inducible lipoprotein | osmB | 3.83 |  | K04062 |
| 2619647233 | Nucleotide-binding universal stress protein, UspA family | uspA | 3.42 | COG0589 |  |
| 2619647880 | Catalase | katE, CAT, catB | 1.95 | COG0753 |  |
| 2619646406 | Catalase | katE, CAT, catB | 1.84 | COG0753 |  |
| 2619646404 | Cytochrome c peroxidase |  | 1.70 | COG1858 |  |
| ***Transcription*** | |  |  |  |  |
| 2619647902 | DNA-binding transcriptional regulator, Nac, LysR family | lysR, nac | 5.19 | COG0583 | K19338 |
| 2619644323 | DNA-binding transcriptional regulator, GntR family | GntR | 3.80 | COG2188 | K03710 |
| 2619646876 | DNA-binding transcriptional regulator, IclR family |  | 2.59 | COG1414 |  |
| 2619645166 | DNA-binding transcriptional regulator, LysR family |  | 2.15 | COG0583 |  |
| 2619646581 | DNA-binding response regulator, NarL/FixJ family, contains REC and HTH domains |  | 1.55 | COG2197 |  |
| ***Transposons*** | |  |  |  |  |
| 2619646664 | Transposase and inactivated derivatives, IS5 family |  | 1.86 | COG3039 | K07481 |
| 2619647232 | Transposase and inactivated derivatives, IS5 family |  | 1.80 | COG3039 | K07481 |
| 2619644443 | Plasmid stabilization system protein ParE |  | 1.74 | COG3668 |  |
| 2619647514 | Transposase and inactivated derivatives, IS5 family |  | 1.70 | COG3039 | K07481 |
| 2619647785 | Transposase and inactivated derivatives, IS5 family |  | 1.63 | COG3039 | K07481 |
| 2619645329 | Transposase and inactivated derivatives, IS5 family |  | 1.60 | COG3039 | K07481 |
