# Supplementary material for: Comparative Transcriptomics of Cold Growth and Adaptive Features of a Eury- and Steno-Psychrophile
Source: Front Microbiol. 2018 Jul 31;9:1565. doi: 10.3389/fmicb.2018.01565 (PMC6080646; doi:10.3389/fmicb.2018.01565)
Supplement: Supplementary file 4 [file Table_4.docx]

***Supplementary Material***

**Comparative Transcriptomics of Cold Growth and Adaptive Features of a Eury- and Steno-psychrophile**

**Isabelle Raymond-Bouchard^1^, Julien Tremblay^2^, Ianina Altshuler^1^, Charles Greer^2^, Lyle G. Whyte^1*^**

***Correspondence**: Lyle Whyte: [lyle.whyte@mcgill.ca](mailto:lyle.whyte@mail.mcgill.ca)

**Supplementary Table 4**

**Table S4**. Additional genes differentially regulated at -5°C compared to 25°C in *Rhodococcus* sp. JG3.

| Gene | Gene Description | Shortname | logFC | COG | KO |
| --- | --- | --- | --- | --- | --- |
| **Amino acid metabolism** | |  |  |  |  |
| 2529301108 | Acetolactate synthase, small subunit | E2.2.1.6S, ilvH, ilvN | 2.75 | 0440 | K01653 |
| 2529299490 | Dihydroxyacid dehydratase/phosphogluconate dehydratase | ilvD | 2.44 | 0129 | K01687 |
| 2529301808 | Glutamate synthase domain 2 | gltB | 2.40 | 0069 | K00265 |
| 2529301109 | Ketol-acid reductoisomerase | ilvC | 2.39 | 0059 | K00053 |
| 2529298629 | ABC-type dipeptide/oligopeptide/nickel transport system, perme | oppC | 2.22 | 1173 | K15582 |
| 2529300014 | 1-aminocyclopropane-1-carboxylate deaminase/D-cysteine desulf | E3.5.99.7 | 2.16 | 2515 | K01505 |
| 2529298751 | Tryptophan 2,3-dioxygenase | E1.13.11.11, TDO2, kynA | 2.14 | 3483 | K00453 |
| 2529299782 | Serine acetyltransferase | cysE | 1.99 | 1045 | K00640 |
| 2529302280 | D-serine deaminase, pyridoxal phosphate-dependent |  | 1.72 | 3616 |  |
| 2529299577 | Threonine dehydrogenase or related Zn-dependent dehydrogenase | SORD, gutB | 1.68 | 1063 | K00008 |
| 2529298750 | Kynureninase | KYNU, kynU | 1.63 | 3844 | K01556 |
| 2529299933 | Alanine dehydrogenase | ald | -4.74 | 0686 | K00259 |
| 2529300105 | 1-pyrroline-5-carboxylate dehydrogenase |  | -4.57 | 1012 | K00294 |
| 2529300636 | Glutamate/leucine/valine dehydrogenase | vdh | -4.34 | 0334 | K00271 |
| 2529298885 | 3-methylcrotonyl-CoA carboxylase beta subunit |  | -4.25 | 4799 | K01969 |
| 2529303137 | Prolyl oligopeptidase PreP, S9A serine peptidase family |  | -2.29 | 1505 | K01322 |
| 2529299129 | 4-aminobutyrate aminotransferase or related aminotransferase |  | -2.24 | 0160 | K03918 |
| 2529299938 | Dipeptidyl aminopeptidase/acylaminoacyl peptidase |  | -2.10 | 1506 |  |
| 2529300603 | Threonine dehydrogenase or related Zn-dependent dehydrogenase |  | -1.87 | 1063 | K00100 |
| 2529302122 | 4-aminobutyrate aminotransferase or related aminotransferase |  | -1.75 | 0160 | K00836 |
| **Carbohydrate transport and metabolism** | |  |  |  |  |
| 2529299881 | Phosphoketolase | xfp, xpk | 4.42 | 3957 | K01621 |
| 2529302279 | 2-dehydro-3-deoxygluconokinase | kdgK | 3.72 | 0524 | K00874 |
| 2529302262 | Glucan/starch phosphorylase | glgP, PYG | 3.67 | 0058 | K00688 |
| 2529300073 | Beta-phosphoglucomutase or related phosphatase, HAD superfamil |  | 3.28 | 0637 |  |
| 2529302373 | Sugar phosphate isomerase/epimerase |  | 3.19 | 1082 |  |
| 2529302770 | Glycosyltransferase, GT2 family |  | 2.92 | 1216 |  |
| 2529301505 | Glyceraldehyde-3-phosphate dehydrogenase/erythrose-4-phosphate | GAPDH, gapA | 1.70 | 0057 | K00134 |
| 2529300360 | Glucose-6-phosphate isomerase | GPI, pgi | 1.56 | 0166 | K01810 |
| 2529299576 | Mannitol-1-phosphate/altronate dehydrogenases | E1.1.1.67, mtlK | 1.57 | 0246 | K00045 |
| 2529298889 | Citrate lyase beta subunit | citE | -5.69 | 2301 | K01644 |
| 2529301342 | 1-phosphofructokinase | fruK, PfkB | -3.50 | 1105 | K00882 |
| 2529299505 | N-acetylglucosaminyl deacetylase, LmbE family |  | -3.39 | 2120 |  |
| 2529303035 | Glucose/arabinose dehydrogenase, beta-propeller fold |  | -3.13 | 2133 |  |
| 2529303109 | D-arabinose 1-dehydrogenase, Zn-dependent alcohol dehydrogenas |  | -2.37 | 1064 | K13979 |
| 2529301213 | Predicted arabinose efflux permease, MFS family |  | -1.79 | 2814 |  |
| 2529298736 | Predicted arabinose efflux permease, MFS family |  | -1.73 | 2814 |  |
| **Cell cycle control, cell division, chromosome partitioning** | |  |  |  |  |
| 2529301194 | Cell division protein FtsI/penicillin-binding protein 2 |  | -5.60 | 0768 |  |
| 2529302119 | Fluoride ion exporter CrcB/FEX, affects chromosome condensatio |  | -2.88 | 0239 | K06199 |
| **Cell wall/membrane/envelope biogenesis** | |  |  |  |  |
| 2529300085 | 2,4-dienoyl-CoA reductase or related NADH-dependent reductase, |  | 4.97 | 1902 |  |
| 2529302896 | Phospholipid/cholesterol/gamma-HCH transport system substrate-binding protein | mlaD | 3.00 | 1463 | **K02067** |
| 2529302895 | Phospholipid/cholesterol/gamma-HCH transport system substrate-binding protein | | 2.60 | COG1463 |  |
| 2529302893 | Phospholipid/cholesterol/gamma-HCH transport system substrate-binding protein | mlaD, linM | 2.45 | 1463 | **K02067** |
| 2529302897 | Phospholipid/cholesterol/gamma-HCH transport system substrate-binding protein | mlaD, linM | 1.61 | 1463 | **K02067** |
| 2529303392 | Apolipoprotein N-acyltransferase | Int | -5.68 | 0815 | K03820 |
| 2529302696 | Membrane-bound lytic murein transglycosylase B |  | -3.08 | 2951 |  |
| 2529301886 | UDP-N-acetylglucosamine--N-acetylmuramyl-(pentapeptide) pyrophosphoryl-undecaprenol N-acetylglucosamine transferase | murG | -3.08 | 0707 | K02563 |
| 2529302604 | Anionic cell wall polymer biosynthesis enzyme, LytR-Cps2A-Psr |  | -2.92 | 1316 |  |
| 2529303387 | Murein DD-endopeptidase MepM and murein hydrolase activator Nl |  | -2.72 | 0739 |  |
| 2529301592 | AAA+-type ATPase, SpoVK/Ycf46/Vps4 family |  | -1.94 | 0464 | K13527 |
| 2529300019 | Glycosyltransferase involved in cell wall bisynthesis |  | -1.53 | 0438 |  |
| **Coenzyme transport and metabolism** | |  |  |  |  |
| 2529300276 | Pimeloyl-ACP methyl ester carboxylesterase |  | 4.77 | 0596 |  |
| 2529302665 | Flavin-dependent oxidoreductase, luciferase family (includes a |  | 4.73 | 2141 |  |
| 2529300146 | Aspartate 1-decarboxylase | panD | 3.11 | 0853 | K01579 |
| 2529301838 | Aspartate oxidase | nadB | 3.06 | 0029 | K00278 |
| 2529299832 | Nicotinamidase-related amidase |  | 3.04 | 1335 |  |
| 2529300234 | Flavin-dependent oxidoreductase, luciferase family (includes a |  | 2.76 | 2141 |  |
| 2529299395 | Flavin-dependent oxidoreductase, luciferase family (includes a |  | 2.55 | 2141 |  |
| 2529301839 | Quinolinate synthase | nadA | 1.81 | 0379 | K03517 |
| 2529302958 | Pimeloyl-ACP methyl ester carboxylesterase |  | 1.74 | 0596 |  |
| 2529300910 | Glutathione synthase/RimK-type ligase, ATP-grasp superfamily |  | 1.57 | 0189 |  |
| 2529300026 | NH3-dependent NAD+ synthetase | nadE | -6.05 | 0171 | K01916 |
| 2529299256 | Flavin-dependent oxidoreductase, luciferase family (includes alkanesulfonate monooxygenase SsuD and methylene tetrahydromethanopterin reductase) | | -4.56 | COG2141 |  |
| 2529301905 | Geranylgeranyl pyrophosphate synthase | idsA | -4.56 | 0142 | K13787 |
| 2529298656 | Flavin-dependent oxidoreductase, luciferase family (includes alkanesulfonate monooxygenase SsuD and methylene tetrahydromethanopterin reductase) | | -3.05 | COG2141 |  |
| 2529300025 | Nicotinamide mononucleotide (NMN) deamidase PncC |  | -3.00 | 1546 | K03743 |
| 2529302712 | Dihydropteroate synthase |  | -2.33 | 0294 | K00796 |
| 2529302107 | Phosphoglycerate dehydrogenase or related dehydrogenase |  | -1.54 | 0111 |  |
| **Defense mechanisms** | |  |  |  |  |
| 2529301356 | Alkyl hydroperoxide reductase subunit AhpC (peroxiredoxin) | E1.11.1.15, PRDX, ahpC | 1.67 | 0450 | K03386 |
| 2529298994 | S-formylglutathione hydrolase FrmB | fbp | 1.67 | 0627 | K18851 |
| 2529302178 | ABC-type multidrug transport system, ATPase and permease compo |  | -2.31 | 1132 | K06147 |
| 2529298479 | Organic hydroperoxide reductase OsmC/OhrA |  | -1.82 | 1764 | K04063 |
| **Energy production and conversion** | |  |  |  |  |
| 2529300679 | Alcohol dehydrogenase, class IV |  | 5.78 | 1454 |  |
| 2529300685 | Anaerobic selenocysteine-containing dehydrogenase |  | 5.77 | 0243 |  |
| 2529300706 | Acyl-CoA reductase or other NAD-dependent aldehyde dehydrogena | aldB | 2.83 | 1012 | K00138 |
| 2529302461 | Acyl-CoA reductase or other NAD-dependent aldehyde dehydrogena | gabD | 2.71 | 1012 | K00135 |
| 2529301126 | Glycerol-3-phosphate dehydrogenase | gpsA | 2.53 | 0240 | K00057 |
| 2529299108 | NADP-dependent 3-hydroxy acid dehydrogenase YdfG |  | 2.37 | 4221 |  |
| 2529303160 | Quinol monooxygenase YgiN |  | 2.28 | 1359 |  |
| 2529300269 | Citrate synthase | CS, gltA | 1.69 | 0372 | K01647 |
| 2529298839 | Glycerol-3-phosphate dehydrogenase | glpA, glpD | 1.63 | 0578 | K00111 |
| 2529302587 | NAD/NADP transhydrogenase alpha subunit | pntA | -5.87 | 3288 | K00324 |
| 2529299416 | Ferredoxin-NADP reductase |  | -5.60 | 1018 |  |
| 2529298812 | Glycolate oxidase | glcD | -3.50 | 0277 | **K00104** |
| 2529299710 | Pyruvate dehydrogenase complex, dehydrogenase (E1) component | aceE | -3.39 | 2609 | K00163 |
| 2529303393 | Cytochrome c biogenesis protein ResB | resB, ccs1 | -3.11 | 1333 | K07399 |
| 2529300639 | Pyruvate/2-oxoglutarate/acetoin dehydrogenase complex, dehydro |  | -2.96 | 0022 | K00162 |
| 2529303388 | Cytochrome c oxidase assembly factor CtaG |  | -2.86 | 3336 | K07245 |
| 2529300192 | ABC-type transport system involved in cytochrome bd biosynthes |  | -2.79 | 4987 | K16012 |
| 2529302653 | FAD/FMN-containing dehydrogenase |  | -2.72 | 0277 | K00103 |
| 2529302644 | CO or xanthine dehydrogenase, Mo-binding subunit |  | -2.43 | 1529 | K11177 |
| 2529300638 | TPP-dependent pyruvate or acetoin dehydrogenase subunit alpha |  | -2.30 | 1071 | K00161 |
| 2529299426 | NADPH:quinone reductase or related Zn-dependent oxidoreductase |  | -2.26 | 0604 | K00344 |
| 2529303224 | Isocitrate/isopropylmalate dehydrogenase |  | -1.97 | 0473 | K07246 |
| 2529302187 | Ferredoxin-NADP reductase |  | -1.94 | 1018 |  |
| 2529298805 | Pyruvate/2-oxoglutarate dehydrogenase complex, dihydrolipoamid |  | -1.73 | 0508 | **K00627** |
| 2529303384 | Heme/copper-type cytochrome/quinol oxidase, subunit 1 |  | -1.72 | 0843 | K02274 |
| 2529300640 | Pyruvate/2-oxoglutarate dehydrogenase complex, dihydrolipoamid |  | -1.67 | 0508 | **K00627** |
| 2529299617 | Phosphoenolpyruvate carboxykinase, GTP-dependent |  | -1.52 | 1274 | K01596 |
| **Inorganic ion transport and metabolism** | |  |  |  |  |
| 2529298548 | Arsenite efflux pump ArsB, ACR3 family |  | 4.73 | 0798 |  |
| 2529301234 | ABC-type molybdate transport system, periplasmic component | modA | 4.73 | 0725 | K02020 |
| 2529300197 | 3-mercaptopyruvate sulfurtransferase SseA, contains two rhodan | sseA, TST, MPST | 3.23 | 2897 | K01011 |
| 2529300708 | Divalent metal cation (Fe/Co/Zn/Cd) transporter |  | 3.04 | 0053 |  |
| 2529302085 | ABC-type sulfate transport system, periplasmic component | cysP | 3.11 | 1613 | K02048 |
| 2529300395 | Mg2+ and Co2+ transporter CorA | corA | 2.01 | 0598 | K03284 |
| 2529300990 | Cyanate permease | MFS.CP | 1.54 | 2807 | K03449 |
| 2529298976 | Membrane protein TerC, possibly involved in tellurium resistan |  | -2.92 | 0861 | K05794 |
| 2529299035 | Ferritin |  | -2.08 | 1528 |  |
| **Intracellular trafficking, secretion, and vesicular transport** | |  |  |  |  |
| 2529301447 | Preprotein translocase subunit SecF | secF | 1.54 | 0341 | K03074 |
| **Lipid transport and metabolism** | |  |  |  |  |
| 2529299636 | Acetyl-CoA acetyltransferase | atoB | 4.90 | 0183 | K00626 |
| 2529299781 | Lysophospholipase L1 or related esterase |  | 2.06 | 2755 |  |
| 2529301248 | 1-acyl-sn-glycerol-3-phosphate acyltransferase | plsC | 1.77 | 0204 | K00655 |
| 2529301920 | Long-chain acyl-CoA synthetase (AMP-forming) | ACSL, fadD | 1.66 | 1022 | K01897 |
| 2529298861 | Formyl-CoA transferase | frc | -4.57 | 1804 | K07749 |
| 2529298818 | Acetyl-CoA acetyltransferase |  | -4.02 | 0183 | **K00680** |
| 2529298819 | Enoyl-CoA hydratase/carnithine racemase |  | -3.95 | 1024 |  |
| 2529298886 | Acetyl/propionyl-CoA carboxylase, alpha subunit | bccA | -3.76 | 4770 | K11263 |
| 2529298887 | Acyl-CoA dehydrogenase related to the alkylation response prot |  | -3.13 | 1960 | K00257 |
| 2529300808 | Choline dehydrogenase or related flavoprotein |  | -2.60 | 2303 | K03333 |
| 2529298888 | Acyl dehydratase |  | -2.39 | 2030 |  |
| 2529301317 | Phosphatidylglycerophosphate synthase |  | -2.18 | 0558 | K00995 |
| 2529299272 | Myo-inositol-1-phosphate synthase |  | -1.92 | 1260 | K01858 |
| 2529301182 | CDP-diglyceride synthetase |  | -1.92 | 0575 | K00981 |
| 2529302186 | Fatty acid desaturase |  | -1.86 | 3239 | K00508 |
| 2529298657 | NAD(P)-dependent dehydrogenase, short-chain alcohol dehydrogen |  | -1.72 | 1028 |  |
| 2529298703 | 3-hydroxyacyl-CoA dehydrogenase |  | -1.59 | 1250 | K01782 |
| 2529302220 | NAD(P)-dependent dehydrogenase, short-chain alcohol dehydrogen |  | -1.58 | 1028 | K00540 |
| 2529298540 | Acyl-CoA dehydrogenase related to the alkylation response prot |  | -1.58 | 1960 | K00249 |
| **Nucleotide transport and metabolism** | |  |  |  |  |
| 2529302300 | ADP-ribose pyrophosphatase YjhB, NUDIX family |  | 3.15 | 1051 |  |
| 2529300862 | Cytidine deaminase | cdd, CDA | 2.78 | 0295 | K01489 |
| 2529301730 | Cytidylate kinase | cmk | 1.90 | 0283 | K00945 |
| 2529302406 | ppGpp synthetase catalytic domain (RelA/SpoT-type nucleotidylt |  | 1.57 | 2357 |  |
| 2529301063 | Ribonucleotide reductase beta subunit, ferritin-like domain |  | -2.12 | 0208 | K00526 |
| 2529301060 | Protein involved in ribonucleotide reduction |  | -1.97 | 1780 | K03647 |
| 2529301061 | Ribonucleotide reductase alpha subunit |  | -1.68 | 0209 | K00525 |
| **Osmoregulation** |  |  |  |  |  |
| 2529301957 | ABC-type proline/glycine betaine transport system, permease co | opuBD | 2.40 | 1174 | **K05846** |
| **Posttranslational modification, protein turnover, chaperones** | |  |  |  |  |
| 2529300820 | Protein-S-isoprenylcysteine O-methyltransferase Ste14 |  | 3.43 | 2020 |  |
| 2529303158 | Metal-sulfur cluster biosynthetic enzyme |  | 3.28 | 2151 |  |
| 2529300799 | Co-chaperonin GroES (HSP10) | groES | 3.25 | 0234 | K04078 |
| 2529303135 | Zn-dependent protease with chaperone function |  | 2.53 | 0501 |  |
| 2529300800 | Chaperonin GroEL (HSP60 family) | groEL, HSPD1 | 2.16 | 0459 | **K04077** |
| 2529303164 | Chaperonin GroEL (HSP60 family) | groEL, HSPD1 | 1.84 | 0459 | **K04077** |
| 2529302154 | ATP-dependent protease ClpP, protease subunit | clpP, CLPP | 1.80 | 0740 | K01358 |
| 2529301523 | Fe-S cluster assembly scaffold protein SufB | sufB | 1.71 | 0719 | K09014 |
| 2529299191 | Peptidyl-prolyl cis-trans isomerase (rotamase) - cyclophilin f | PPIA | 1.70 | 0652 | K03767 |
| 2529303394 | ABC-type transport system involved in cytochrome c biogenesis, |  | -5.83 | 0755 |  |
| 2529300021 | Gamma-glutamyl:cysteine ligase YbdK, ATP-grasp superfamily | ybdK | -5.36 | 2170 | K06048 |
| 2529302519 | ADP-ribosylglycohydrolase |  | -5.19 | 1397 |  |
| 2529301852 | NAD-dependent protein deacetylase, SIR2 family |  | -2.98 | 0846 | K12410 |
| 2529303174 | Thioredoxin reductase |  | -2.57 | 0492 | K00384 |
| 2529299466 | ATP-dependent Lon protease, bacterial type |  | -2.53 | 0466 | K01338 |
| 2529302124 | NAD-dependent protein deacetylase, SIR2 family |  | -1.58 | 0846 |  |
| **Replication, recombination and repair** | |  |  |  |  |
| 2529302415 | Predicted ATP-dependent endonuclease of the OLD family, contai |  | 1.89 | 3593 |  |
| 2529302709 | 3-methyladenine DNA glycosylase Tag |  | -2.21 | 2818 | K01246 |
| **Secondary metabolites biosynthesis, transport and catabolism** | |  |  |  |  |
| 2529299331 | Catechol 2,3-dioxygenase or other lactoylglutathione lyase fam |  | 5.62 | 0346 |  |
| 2529298397 | Taurine dioxygenase, alpha-ketoglutarate-dependent | tauD | 5.53 | 2175 | K03119 |
| 2529300596 | 2-keto-4-pentenoate hydratase/2-oxohepta-3-ene-1,7-dioic acid |  | 4.35 | 0179 |  |
| 2529300141 | Non-ribosomal peptide synthetase component F |  | 3.84 | 1020 |  |
| 2529299792 | Isopenicillin N synthase and related dioxygenases |  | 2.84 | 3491 |  |
| 2529299150 | Phenolic acid decarboxylase | pdc | 2.75 | 3479 | K13727 |
| 2529300142 | Non-ribosomal peptide synthetase component F |  | 2.14 | 1020 |  |
| 2529298797 | Homogentisate 1,2-dioxygenase | HGD, hmgA | 1.88 | 3508 | K00451 |
| 2529301617 | Catechol 2,3-dioxygenase or other lactoylglutathione lyase fam |  | 1.75 | 0346 |  |
| 2529298824 | 1,2-phenylacetyl-CoA epoxidase, catalytic subunit |  | -2.38 | 3396 | K02611 |
| 2529298814 | Acyl-coenzyme A thioesterase PaaI, contains HGG motif |  | -2.15 | 2050 | K02614 |
| 2529299513 | Cytochrome P450 |  | -2.10 | 2124 | K00517 |
| **Signal transduction mechanisms** | |  |  |  |  |
| 2529300535 | Signal transduction histidine kinase |  | 4.80 | 0642 |  |
| 2529299858 | EAL domain, c-di-GMP-specific phosphodiesterase class I (or it |  | 2.74 | 2200 |  |
| 2529299877 | DNA-binding response regulator, NarL/FixJ family, contains REC | devR | 2.32 | 2197 | K07695 |
| 2529301683 | Forkhead associated (FHA) domain, binds pSer, pThr, pTyr |  | 1.82 | 1716 |  |
| 2529301153 | Nitrogen regulatory protein PII | glnB | 1.75 | 0347 | K04751 |
| 2529299483 | Adenylate cyclase, class 3 | E4.6.1.1 | 1.69 | 2114 | K01768 |
| 2529300020 | Anti-anti-sigma regulatory factor (antagonist of anti-sigma fa |  | -4.24 | 1366 |  |
| 2529300028 | Anti-anti-sigma regulatory factor (antagonist of anti-sigma fa |  | -1.86 | 1366 |  |
| 2529299913 | Anti-anti-sigma regulatory factor (antagonist of anti-sigma fa |  | -1.62 | 1366 |  |
| 2529300101 | Anti-anti-sigma regulatory factor (antagonist of anti-sigma fa |  | -1.61 | 1366 |  |
| **Transcription** | |  |  |  |  |
| 2529303125 | DNA-binding transcriptional regulator, XRE-family HTH domain |  | 5.02 | 1476 | K07729 |
| 2529298833 | DNA-binding transcriptional regulator, MarR family |  | 2.96 | 1846 |  |
| 2529302323 | DNA-binding transcriptional regulator, ArsR family | arsR | 2.75 | 0640 | K03892 |
| 2529300496 | DNA-binding transcriptional regulator, LysR family |  | 2.66 | 0583 |  |
| 2529299074 | DNA-binding transcriptional regulator, AcrR family |  | 2.51 | 1309 |  |
| 2529302950 | DNA-binding transcriptional regulator, AcrR family |  | 2.27 | 1309 |  |
| 2529298898 | DNA-binding transcriptional regulator, LysR family |  | 2.18 | 0583 |  |
| 2529299633 | DNA-binding transcriptional regulator, AcrR family |  | 2.01 | 1309 |  |
| 2529300506 | DNA-binding transcriptional regulator, LysR family |  | 1.94 | 0583 |  |
| 2529302580 | DNA-binding transcriptional regulator, GntR family | K03710 | 1.88 | 2188 | K03710 |
| 2529298519 | DNA-binding transcriptional regulator, GntR family |  | 1.68 | 1802 |  |
| 2529299557 | DNA-binding transcriptional regulator, AcrR family |  | -5.36 | 1309 |  |
| 2529298828 | AraC-type DNA-binding domain and AraC-containing proteins |  | -4.58 | 2207 |  |
| 2529298884 | DNA-binding transcriptional regulator, AcrR family |  | -2.75 | 1309 |  |
| 2529299273 | DNA-binding transcriptional regulator, PadR family |  | -2.40 | 1695 |  |
| 2529302179 | DNA-binding transcriptional regulator, MarR family |  | -2.27 | 1846 |  |
| 2529301343 | DNA-binding transcriptional regulator of sugar metabolism, Deo |  | -2.19 | 1349 | K03436 |
| 2529298615 | Cold shock protein, CspA family |  | -2.18 | 1278 | K03704 |
| 2529302703 | DNA-directed RNA polymerase specialized sigma subunit, sigma24 |  | -1.91 | 1595 | **K03088** |
| 2529302008 | Cold shock protein, CspA family |  | -1.64 | 1278 |  |
| 2529299115 | DNA-binding transcriptional regulator, ArsR family |  | -1.63 | 0640 |  |
| **Translation, ribosomes, helicases** | |  |  |  |  |
| 2529301179 | Translation elongation factor EF-Ts | tsf, TSFM | 1.87 | 0264 | K02357 |
| 2529300770 | Ribosomal protein L17 | RP-L17, MRPL17, rplQ | 1.84 | 0203 | K02879 |
| 2529299296 | Ribosomal protein S6 | RP-S6, MRPS6, rpsF | 1.77 | 0360 | K02990 |
| 2529302903 | Ribosomal protein L10 | RP-L10, MRPL10, rplJ | 1.71 | 0244 | K02864 |
| 2529301875 | Isoleucyl-tRNA synthetase | IARS, ileS | 1.66 | 0060 | K01870 |
| 2529300767 | Ribosomal protein S11 | RP-S11, MRPS11, rpsK | 1.65 | 0100 | K02948 |
| 2529300383 | Ribosomal protein S18 | RP-S18, MRPS18, rpsR | 1.63 | 0238 | K02963 |
| 2529299299 | Ribosomal protein L9 | RP-L9, MRPL9, rplI | 1.57 | 0359 | K02939 |
| 2529300754 | Ribosomal protein S8 | RP-S8, rpsH | 1.56 | 0096 | K02994 |
| 2529301767 | Ribosomal protein L20 | RP-L20, MRPL20, rplT | 1.55 | 0292 | K02887 |
| 2529300738 | Ribosomal protein S19 | RP-S19, rpsS | 1.53 | 0185 | K02965 |
| 2529301332 | tRNA A37 methylthiotransferase MiaB |  | -1.53 | 0621 | K06168 |
