# Supplementary material for: Comparative Transcriptomics of Cold Growth and Adaptive Features of a Eury- and Steno-Psychrophile
Source: Front Microbiol. 2018 Jul 31;9:1565. doi: 10.3389/fmicb.2018.01565 (PMC6080646; doi:10.3389/fmicb.2018.01565)
Supplement: Supplementary file 5 [file Table_5.docx]

***Supplementary Material***

**Comparative Transcriptomics of Cold Growth and Adaptive Features of a Eury- and Steno-psychrophile**

**Isabelle Raymond-Bouchard^1^, Julien Tremblay^2^, Ianina Altshuler^1^, Charles Greer^2^, Lyle G. Whyte^1*^**

***Correspondence**: Lyle Whyte: [lyle.whyte@mcgill.ca](mailto:lyle.whyte@mail.mcgill.ca)

**Supplementary Table 5**

**Table S5**. Additional genes differentially regulated at 0°C compared to 20°C in *Polaromonas* sp. Eur3 1.2.1.

| **Gene** | **Gene Description** | **Shortname** | **logFC** | **COG** | **KO** |
| --- | --- | --- | --- | --- | --- |
| **Amino acid transport and metabolism** | |  |  |  |  |
| 2619646948 | Glutamate dehydrogenase/leucine dehydrogenase | gdhA | 1.79 | COG0334 | K00261 |
| 2619646216 | Kynurenine formamidase |  | 1.68 | COG1878 |  |
| 2619647247 | Archaeal aspartate aminotransferase or a related aminotransferase, includes purine catabolism protein PucG | | 1.56 | COG0075 |  |
| 2619647679 | oxalyl-CoA decarboxylase | oxc | -3.96 | COG0028 | K01577 |
| 2619643647 | ATP phosphoribosyltransferase regulatory subunit HisZ | hisZ | -2.58 | COG3705 | K02502 |
| 2619645801 | Xaa-Pro aminopeptidase | pepP | -2.32 | COG0006 | K01262 |
| 2619643644 | Cystathionine beta-lyase/cystathionine gamma-synthase | metC | -2.12 | COG0626 | K01760 |
| 2619645859 | 3-deoxy-D-arabino-heptulosonate 7-phosphate (DAHP) synthase | aroF, aroG, aroH | -2.01 | COG0722 | K01626 |
| 2619647674 | NADPH-dependent glutamate synthase beta chain or related oxidoreductase | fdoG, fdfH | -1.96 | COG0493 | K00123 |
| 2619645815 | Acetylornithine deacetylase/Succinyl-diaminopimelate desuccinylase or related deacylase | | -1.92 | COG0624 |  |
| 2619646235 | ABC-type amino acid transport system, permease component | ABC.PA.P | -1.78 | COG0765 | K02029 |
| 2619644380 | Argininosuccinate lyase | argH | -1.54 | COG0165 | K01755 |
| **Carbohydrate transport and metabolism** | |  |  |  |  |
| 2619644232 | Phosphomannomutase | manB | -1.61 | COG1109 | K01840 |
| 2619645656 | Pyruvate kinase | pyk | -1.56 | COG0469 | K00873 |
| **Cell cycle control, cell division, chromosome partitioning** | |  |  |  |  |
| 2619644948 | GTP-binding protein EngB required for normal cell division | engB | -2.19 | COG0218 | K03978 |
| **Cell motility** | |  |  |  |  |
| 2619643841 | Flp pilus assembly protein, secretin CpaC | cpaC, rcpA | -6.10 | COG4964 | K02280 |
| 2619643846 | Flp pilus assembly protein, pilin Flp | flp, pilA | -4.20 | COG3847 | K02651 |
| 2619643845 | Flp pilus assembly protein, pilin Flp | flp, pilA | -4.09 | COG3847 | K02651 |
| **Cell wall/membrane/envelope biogenesis** | |  |  |  |  |
| 2619644849 | D-alanine-D-alanine ligase and related ATP-grasp enzymes | cphA | 1.75 | COG1181 | K03802 |
| 2619646061 | ABC-type transporter Mla maintaining outer membrane lipid asymmetry, periplasmic component MlaD | mlaD, linM | 1.54 | COG1463 | K02067 |
| 2619644269 | Outer membrane protein OmpA and related peptidoglycan-associated (lipo)proteins | | 1.50 | COG2885 |  |
| 2619646018 | Alanine racemase | alr | -5.24 | COG0787 | K01775 |
| 2619645404 | Putative effector of murein hydrolase |  | -5.12 | COG1346 |  |
| 2619643705 | Lipid A disaccharide synthetase | lpxB | -4.34 | COG0763 | K00748 |
| 2619645251 | dTDP-4-dehydrorhamnose 3,5-epimerase | rfbC | -2.81 | COG1898 | K01790 |
| 2619645698 | O-antigen ligase |  | -2.41 | COG3307 |  |
| 2619646670 | Outer membrane translocation and assembly module TamA | tamA | -2.24 | COG0729 | K07278 |
| 2619643706 | Acyl-[acyl carrier protein]--UDP-N-acetylglucosamine O-acyltransferase | lpxA | -2.23 | COG1043 | K00677 |
| 2619644424 | Glycosyltransferase involved in cell wall bisynthesis |  | -1.69 | COG0438 |  |
| 2619646077 | D-alanyl-D-alanine carboxypeptidase | dacC, dacA, dacD | -1.67 | COG1686 | K07258 |
| **Coenzyme transport and metabolism** | |  |  |  |  |
| 2619646403 | Non-heme chloroperoxidase | cpo | 2.03 | COG0596 | K00433 |
| 2619647644 | Molybdopterin synthase sulfur carrier subunit | moaD | -3.63 | COG1977 | K03636 |
| 2619646734 | Riboflavin kinase / FMN adenylyltransferase | ribF | -2.29 | COG0196 | K11753 |
| 2619646633 | 2-polyprenyl-6-methoxyphenol hydroxylase and related FAD-dependent oxidoreductases | | -2.21 | COG0654 |  |
| 2619646694 | Pimeloyl-ACP methyl ester carboxylesterase |  | -2.14 | COG0596 |  |
| 2619647643 | Molybdopterin molybdotransferase | moeA | -2.03 | COG0303 | K03750 |
| 2619645550 | Glutathione synthase/RimK-type ligase, ATP-grasp superfamily | gshB | -1.73 | COG0189 | K01920 |
| 2619644467 | NAD(P)H-flavin reductase | ascD, ddhD, rfbl | -1.55 | COG0543 | K00523 |
| **Defense mechanisms** | |  |  |  |  |
| 2619643978 | type I restriction enzyme, S subunit | hsdS | 2.23 |  | K01154 |
| 2619643980 | Type I site-specific restriction endonuclease, part of a restriction-modification system | hsdR | 1.72 | COG4096 | K01153 |
| 2619645699 | mRNA interferase MazF | mazF | -2.50 | COG2337 | K07171 |
| 2619643826 | DNA-damage-inducible protein J | dinJ | -2.21 | COG3077 | K07473 |
| 2619645700 | Antitoxin MazE of the MazEF toxin-antitoxin module | mazE, chpAI | -2.42 | COG2336 | K07172 |
| 2619645910 | Plasmid maintenance system killer protein | higA | -1.79 | COG3549 | K07334 |
| 2619646234 | Enamine deaminase RidA, house cleaning of reactive enamine intermediates, YjgF/YER057c/UK114 family | | -1.64 | COG0251 |  |
| **Energy production and conversion** | |  |  |  |  |
| 2619644108 | Cytochrome c551/c552 | CYC | 1.94 | COG4654 | K08738 |
| 2619646935 | NADH:ubiquinone oxidoreductase 24 kD subunit (chain E) | nuoE | 1.93 | COG1905 | K00334 |
| 2619644324 | Succinate dehydrogenase/fumarate reductase, cytochrome b subunit | sdhC, frdC | 1.78 | COG2009 | K00241 |
| 2619646939 | Formate hydrogenlyase subunit 6/NADH:ubiquinone oxidoreductase 23 kD subunit (chain I) | nuoI | 1.75 | COG1143 | K00338 |
| 2619646934 | NADH:ubiquinone oxidoreductase 49 kD subunit (chain D) | nuoD | 1.72 | COG0649 | K00333 |
| 2619645466 | FoF1-type ATP synthase, membrane subunit c/Archaeal/vacuolar-type H+-ATPase, subunit K | ATPF0C, atpE | 1.69 | COG0636 | K02110 |
| 2619646938 | NADH:ubiquinone oxidoreductase subunit 1 (chain H) | nuoH | 1.67 | COG1005 | K00337 |
| 2619646943 | NADH:ubiquinone oxidoreductase subunit 4 (chain M) | nuoM | 1.67 | COG1008 | K00342 |
| 2619645461 | FoF1-type ATP synthase, beta subunit | ATPF1B, atpD | 1.66 | COG0055 | K02112 |
| 2619645462 | FoF1-type ATP synthase, gamma subunit | ATPF1G, atpG | 1.64 | COG0224 | K02115 |
| 2619646411 | Cytochrome c | tsdA | 1.56 | COG3258 | K19713 |
| 2619646937 | NADH dehydrogenase/NADH:ubiquinone oxidoreductase 75 kD subunit (chain G) | nuoG | 1.54 | COG1034 | K00336 |
| 2619646933 | NADH:ubiquinone oxidoreductase 27 kD subunit (chain C) | nuoC | 1.53 | COG0852 | K00332 |
| 2619645803 | Rubredoxin |  | -5.84 | COG1773 |  |
| 2619645601 | (Methyl)Malonate-semialdehyde dehydrogenase (acetylating) | mmsA, iolA | -5.59 | COG1012 | K00140 |
| 2619644823 | Phosphoglycolate phosphatase, HAD superfamily | gph | -5.41 | COG0546 | K01091 |
| 2619647336 | FAD/FMN-containing dehydrogenase |  | -4.37 | COG0277 |  |
| 2619647684 | Tripartite-type tricarboxylate transporter, receptor component TctC | | -4.11 | COG3181 |  |
| 2619646490 | Formate dehydrogenase iron-sulfur subunit | fdoH | -3.35 | COG0437 | K00124 |
| 2619647673 | Formate dehydrogenase alpha subunit | fdhA1 | -2.84 | COG3383 | K05299 |
| 2619646693 | Tripartite-type tricarboxylate transporter, receptor component TctC | | -2.30 | COG3181 |  |
| 2619645505 | Malate synthase | aceB, glcB | -2.22 | COG2225 | K01638 |
| 2619646097 | Cytochrome b |  | -2.09 | COG3658 |  |
| 2619645905 | FAD/FMN-containing dehydrogenase | dld | -1.95 | COG0277 | K00102 |
| 2619646075 | (2Fe-2S) ferredoxin |  | -1.66 | COG3411 |  |
| 2619646450 | Anaerobic selenocysteine-containing dehydrogenase |  | -1.59 | COG0243 |  |
| 2619643817 | Uncharacterized conserved protein |  | -1.51 | COG3019 |  |
| 2619644971 | Uncharacterized conserved protein YbjT, contains NAD(P)-binding and DUF2867 domains | | -1.94 | COG0702 | K00356 |
| 2619645655 | Membrane-associated enzyme, PAP2 (acid phosphatase) superfamily | | -1.84 | COG3907 |  |
| 2619646324 | Predicted dinucleotide-utilizing enzyme | nadX | -1.68 | COG1712 | K06989 |
| **Inorganic ion transport and metabolism** | |  |  |  |  |
| 2619645596 | Mg2+ and Co2+ transporter CorA | corA | 1.61 | COG0598 | K03284 |
| 2619645019 | Magnesium-transporting ATPase (P-type) | E3.6.3.2, mgtA, mgtB | 1.60 | COG0474 | K01531 |
| 2619645583 | Cu2+-exporting ATPase | copB | -6.71 | COG2217 | K01533 |
| 2619647685 | MFS transporter, OFA family, oxalate/formate antiporter | oxlT | -3.90 | COG2223 | K08177 |
| 2619645690 | Membrane protein TerC, possibly involved in tellurium resistance | | -3.07 | COG0861 |  |
| 2619645724 | Membrane protein TerC, possibly involved in tellurium resistance | | -2.48 | COG0861 |  |
| 2619645204 | Carbonic anhydrase | cynT, can | -2.19 | COG0288 | K01673 |
| 2619645159 | ABC-type phosphate/phosphonate transport system, periplasmic component | phnD | -1.84 | COG3221 | K02044 |
| **Intracellular trafficking, secretion, and vesicular transport** | |  |  |  |  |
| 2619647110 | Type II secretory pathway component GspD/PulD (secretin) | gspD | 1.62 | COG1450 | K02453 |
| 2619644574 | Signal recognition particle GTPase | ftsY | -1.65 | COG0552 | K03110 |
| **Lipid transport and metabolism** | |  |  |  |  |
| 2619647004 | 3-hydroxyacyl-CoA dehydrogenase | paaH, hbd, fadB, mmgB | 1.51 | COG1250 | K00074 |
| 2619647678 | Formyl-CoA transferase | frc | -4.95 | COG1804 | K07749 |
| 2619647774 | Acetyl-CoA acetyltransferase | atoB | -2.83 | COG0183 | K00626 |
| 2619647676 | Formyl-CoA transferase | frc | -2.77 | COG1804 | K07749 |
| 2619646556 | Glycerophosphoryl diester phosphodiesterase | glpQ, ugpQ | -2.42 | COG0584 | K01126 |
| 2619647138 | Phytoene/squalene synthetase |  | -2.21 | COG1562 |  |
| 2619647618 | Acyl carrier protein |  | -1.52 | COG0236 |  |
| 2619647425 | Acyl-CoA thioesterase FadM | ybgC | -1.50 | COG0824 | K07107 |
| **Mobilome: prophages, transposons** | |  |  |  |  |
| 2619644208 | comEA; competence protein ComEA | comEA | 1.65 |  | K02237 |
| 2619646169 | Transposase |  | -2.66 | COG3436 |  |
| 2619645345 | Transposase |  | -2.26 | COG3436 |  |
| 2619647498 | Transposase |  | -2.11 | COG3436 |  |
| 2619647190 | Transposase |  | -2.01 | COG3436 |  |
| 2619644154 | Transposase |  | -1.99 | COG3436 |  |
| 2619646282 | Transposase (or an inactivated derivative) |  | -1.87 | COG3316 |  |
| 2619645851 | Transposase |  | -1.86 | COG3436 |  |
| 2619645352 | Transposase |  | -1.81 | COG3436 |  |
| 2619646176 | Transposase |  | -1.76 | COG3436 |  |
| 2619643799 | Transposase |  | -1.62 | COG3436 |  |
| **Nucleotide transport and metabolism** | |  |  |  |  |
| 2619646419 | Nucleoside phosphorylase | amn | 1.98 | COG0775 | K01241 |
| 2619646615 | Ribonucleotide-diphosphate reductase alpha subunit | nrdA, nrdE | -2.14 | COG0209 | K00525 |
| *Other transporters* | |  |  |  |  |
| 2619646708 | ABC-type protease/lipase transport system, ATPase and permease components | hasD, prtD, aprD | -6.70 | COG4618 | K12536 |
| 2619645428 | MFS transporter, DHA1 family, bicyclomycin/chloramphenicol resistance protein | bcr | -2.36 | COG2814 | K07552 |
| **Posttranslational modification, protein turnover, chaperones** | |  |  |  |  |
| 2619647143 | FKBP-type peptidyl-prolyl cis-trans isomerase (trigger factor) | tig | 1.64 | COG0544 | K03545 |
| 2619647912 | ATP-dependent Lon protease, bacterial type | lon | -5.71 | COG0466 | K01338 |
| 2619647792 | Collagenase-like protease, PrtC family |  | -3.72 | COG0826 | K08303 |
| 2619646606 | Thiol-disulfide isomerase or thioredoxin |  | -2.54 | COG0526 |  |
| 2619644737 | Molecular chaperone IbpA, HSP20 family |  | -2.28 | COG0071 |  |
| 2619644738 | Molecular chaperone IbpA, HSP20 family |  | -2.18 | COG0071 | K13993 |
| 2619647767 | Membrane protein implicated in regulation of membrane protease activity | | -1.93 | COG1585 |  |
| 2619645681 | Glutamine synthetase adenylyltransferase | glnE | -1.91 | COG1391 | K00982 |
| 2619644385 | Periplasmic serine protease, S1-C subfamily, contain C-terminal PDZ domain | degP, htrA | -1.67 | COG0265 | K04771 |
| 2619646309 | ABC-type glutathione transport system ATPase component, contains duplicated ATPase domain | | -1.64 | COG1123 | K02032 |
| 2619646095 | Peroxiredoxin |  | -1.51 | COG0678 |  |
| **Replication, recombination and repair** |  |  |  |  |  |
| 2619646142 | rpoB; DNA-directed RNA polymerase subunit beta [EC:2.7.7.6] | rpoB | 1.63 |  | K03043 |
| 2619645631 | DNA polymerase | dpo | -5.44 | COG1573 | K02334 |
| 2619645299 | ATP-dependent DNA helicase Rep | rep | -2.13 | COG0210 | K03656 |
| **Secondary metabolites biosynthesis, transport and catabolism** | |  |  |  |  |
| 2619646842 | 2-keto-4-pentenoate hydratase/2-oxohepta-3-ene-1,7-dioic acid hydratase (catechol pathway) | | 1.81 | COG0179 |  |
| 2619646695 | 2-keto-4-pentenoate hydratase/2-oxohepta-3-ene-1,7-dioic acid hydratase (catechol pathway) | | -3.12 | COG0179 |  |
| 2619645902 | 2-keto-4-pentenoate hydratase/2-oxohepta-3-ene-1,7-dioic acid hydratase (catechol pathway) | | -2.42 | COG0179 |  |
| **Signal transduction mechanisms** | |  |  |  |  |
| 2619647877 | Adenylate cyclase, class 3 |  | 1.88 | COG2114 |  |
| 2619647021 | CBS domain |  | 1.82 | COG0517 |  |
| 2619646291 | Two-component response regulator, PleD family, consists of two REC domains and a diguanylate cyclase (GGDEF) domain | | 1.65 | COG3706 |  |
| 2619646287 | RecA-superfamily ATPase, KaiC/GvpD/RAD55 family | kaiC | 1.52 | COG0467 | K08482 |
| 2619647648 | DNA-binding transcriptional response regulator, NtrC family, contains REC, AAA-type ATPase, and a Fis-type DNA-binding domains | zraR, hydG | 1.51 | COG2204 | K07713 |
| 2619645472 | Putative lipoic acid-binding regulatory protein |  | -5.88 | COG2921 | K09158 |
| 2619647655 | Periplasmic catabolite regulation protein CreA (function unknown) | creA | -2.96 | COG3045 | K05805 |
| 2619645734 | Two-component system, OmpR family, sensor histidine kinase TctE | tctE | -2.93 | COG0642 | K07649 |
| 2619646363 | Ser/Thr protein kinase RdoA involved in Cpx stress response, MazF antagonist | | -1.93 | COG2334 |  |
| 2619644878 | cAMP-binding domain of CRP or a regulatory subunit of cAMP-dependent protein kinases | fnr | -1.81 | COG0664 | K01420 |
| **Stress and oxidative responses** | |  |  |  |  |
| 2619647313 | Nucleotide-binding universal stress protein, UspA family | | -5.54 | COG0589 |  |
| 2619644545 | Nucleotide-binding universal stress protein, UspA family | | -2.38 | COG0589 |  |
| **Transcription** |  |  |  |  |  |
| 2619645768 | Fic family protein |  | -5.61 | COG3177 |  |
| 2619643611 | DNA-binding transcriptional regulator, LacI/PurR family | lacI, galR | -5.24 | COG1609 | K02529 |
| 2619645766 | DNA-binding transcriptional regulator YiaG, XRE-type HTH domain | | -2.61 | COG2944 |  |
| 2619645955 | DNA-binding transcriptional regulator, GntR family |  | -1.69 | COG1802 |  |
| 2619644174 | DNA-binding transcriptional regulator, GntR family |  | -1.58 | COG1802 |  |
| **Translation, ribosomal structure and biogenesis** | |  |  |  |  |
| 2619646087 | Ribosomal protein S19 | RP-S19, rpsS | 1.94 | COG0185 | K02965 |
| 2619646078 | Ribosomal protein S12 | RP-S12, MRPS12, rpsL | 1.82 | COG0048 | K02950 |
| 2619647845 | Ribonuclease G | rng, cafA | 1.76 | COG1530 | K08301 |
| 2619645368 | Ribosomal protein S8 | RP-S8, rpsH | 1.72 | COG0096 | K02994 |
| 2619645366 | Ribosomal protein L18 | RP-L18, MRPL18, rplR | 1.66 | COG0256 | K02881 |
| 2619645367 | Ribosomal protein L6P/L9E | RP-L6, MRPL6, rplF | 1.59 | COG0097 | K02933 |
| 2619645218 | Glycyl-tRNA synthetase, alpha subunit | glyQ | 1.59 | COG0752 | K01878 |
| 2619646090 | Ribosomal protein L16/L10AE | RP-L16, MRPL16, rplP | 1.57 | COG0197 | K02878 |
| 2619644634 | Peptide chain release factor RF-3 | prfC | -2.75 | COG4108 | K02837 |
| 2619647275 | tRNA-dihydrouridine synthase | dusA | -2.10 | COG0042 | K05539 |
| 2619644240 | Ribonuclease E | rne | -1.99 | COG1530 | K08300 |
| 2619647870 | Peptide deformylase | PDF, def | -1.81 | COG0242 | K01462 |
| 2619645150 | Ribosomal protein L13 | RP-L13, MRPL13, rplM | -1.54 | COG0102 | K02871 |
