# Supplementary material for: Comparative Transcriptomics of Cold Growth and Adaptive Features of a Eury- and Steno-Psychrophile
Source: Front Microbiol. 2018 Jul 31;9:1565. doi: 10.3389/fmicb.2018.01565 (PMC6080646; doi:10.3389/fmicb.2018.01565)
Supplement: Supplementary file 6 [file Image_1.pdf]

Supplementary Material

Comparative Transcriptomics of Cold Growth and Adaptive Features of a Eury- and Steno-psychrophile

Isabelle Raymond-Bouchard[1], Julien Tremblay[2], Ianina Altshuler[1], Charles Greer[2], Lyle G. Whyte[1*]

***Correspondence**: Lyle Whyte: lyle.whyte@mcgill.ca

Supplementary Figure

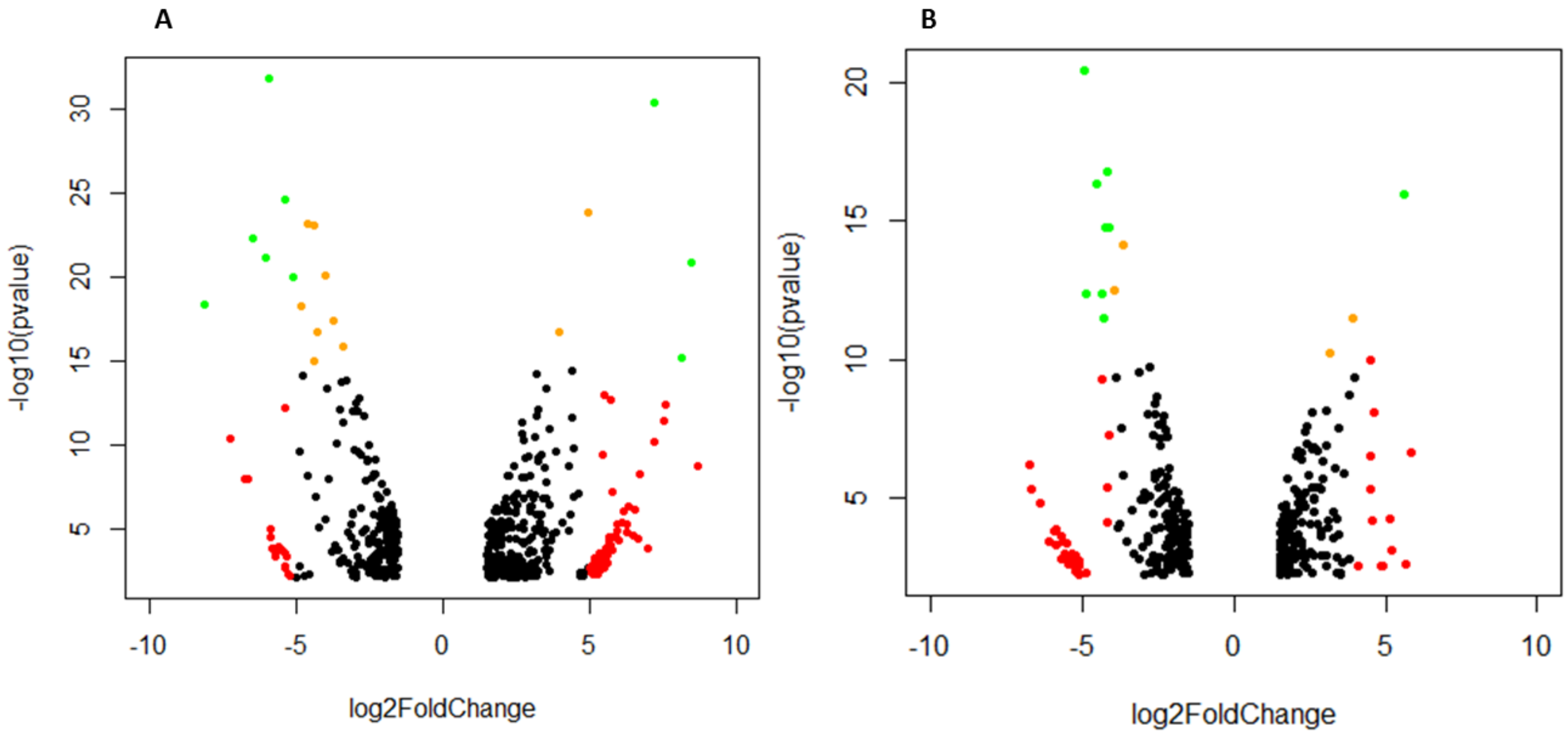

Figure S1. Volcano plot showing differentially expressed genes (log2 fold change) at low temperatures that are statistically significant ($p < 0.05$) in: **A)** *Rhodococcus* sp. JG3 and **B)** *Polaromonas* sp. Eur3 1.2.1. Negative log 2-fold change are downregulated at low temperatures, while positive points represent upregulated genes, as compared to the higher temperatures. Red dots represent expressed genes with log2 fold changes > 5 for *Rhodococcus* sp. JG3 and > 4 for *Polaromonas* sp. Eur3 1.2.1 at the cold temperatures; orange dots represent gene transcripts at cold temperatures with highly significant fold changes, -log10(pvalue) > 15 for *Rhodococcus* sp. JG3 and > 10 for *Polaromonas* sp. Eur3; and green dots represent genes that correspond to both categories.
